# Cs-Bentonite Clay for Biogas Upgrading: A Numerical Assessment

**DOI:** 10.1021/acs.iecr.4c04491

**Published:** 2025-04-10

**Authors:** Niels Mendel, Jordanus J. P. Jordi Boon, Igor Sîreţanu, Frieder Mugele, Derk W. F. Wim Brilman

**Affiliations:** † Physics of Complex Fluids, Faculty of Science and Technology, MESA+ Institute for Nanotechnology, University of Twente, P.O. Box 217, 7500 AE Enschede, The Netherlands; ‡ Sustainable Process Technology, Faculty of Science and Technology, University of Twente, P.O. Box 217, 7500 AE Enschede, The Netherlands

## Abstract

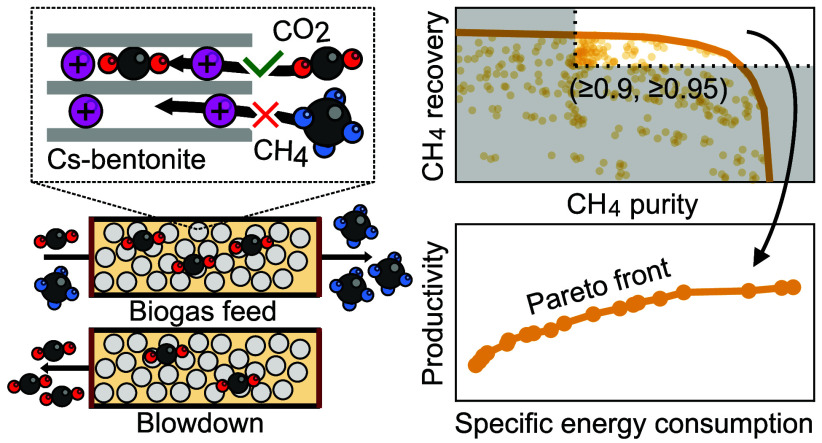

Biogas upgrading by vacuum-pressure swing adsorption involves the selective adsorption of CO_2_ over CH_4_ on a sorbent material to separate both components. This work assesses numerically the performance of the previously characterized Cs-exchanged bentonite clay for this separation. This benchmarking study includes the effect of the process cycle configuration (seven different configurations using one stage and up to three columns), the ambient temperature (15 or 25 °C), the feed biogas composition (CO_2_ mole fraction of 0.35 or 0.45, balance CH_4_), and the process operating parameters. Specific constraints on CH_4_ purity and CH_4_ recovery provide Pareto fronts for maximum productivity and minimum specific energy consumption. A two-column unit operated at ambient feed pressure can upgrade 0.097 Nm^3^ feed biogas (CO_2_ mole fraction of 0.45, balance CH_4_) per kg sorbent per h to a bio-CH_4_ product with a purity of 0.906 and with a CH_4_ recovery of 0.967 at a comparatively low specific energy consumption of only 0.072 kWh per produced Nm^3^ of CH_4_. Using more columns and pressure equalization steps further enhances the CH_4_ recovery. The low bentonite cost, the comparatively low specific energy consumption due to the favorable linear CO_2_ adsorption isotherms, and the high recovery due to the high CO_2_/CH_4_ selectivity make Cs-bentonite an excellent alternative for conventional sorbent materials.

## 1 Introduction

Biogas is a gas mixture produced by the anaerobic digestion of organic matter (organic waste, wastewater sludge, manure, landfill waste, etc.). It comprises mainly CH_4_ (mole fraction of 0.50–0.70) and CO_2_ (mole fraction of 0.30–0.50), as well as other minor contaminants like H_2_S and H_2_O.^1−3^ Separation of the CH_4_ and CO_2_ into a bio-CH_4_ stream that can be used as a natural gas substitute (e.g., for gas grid injection) and a high-purity CO_2_ stream (e.g., used for plant or algae cultivation, chemical production, or sequestration) increases the value of and number of use cases for biogas.^1−5^ This separation is known as biogas upgrading.

Established methods for biogas upgrading include absorption (water, chemical, or physical scrubbing), membrane separation, and ((vacuum) pressure and/or temperature swing) adsorption.^1−7^ All of these methods have their (dis)advantages.^2−6^ However, the adsorption method has been considered of interest because of the compactness of the equipment, low energy consumption and capital costs, safety, and ability to achieve high purification levels.^2−4,8,9^ The adsorption method is a cyclic process. In short, for biogas upgrading, it requires first the selective adsorption of CO_2_ on an adsorbent material, thereby producing high-purity CH_4_. Subsequently, it requires the regeneration of the adsorbent material at reduced (CO_2_ partial) pressure and/or elevated temperature, thereby producing high-purity CO_2_. Therefore, the performance of this method depends strongly on the adsorbent material that is used.^3,5,7,10−14^ Conventional adsorbents used or studied for biogas upgrading include activated carbons (AC),^12,15−21^ various zeolites,^9,10,13,16,22−36^ carbon molecular sieves (CMS),^10−12,14,25,30,32,37−43^ and various other materials.^8,10,16,28,44−50^Table S1 presents performance indicators (if available) from previous works on these different sorbent materials.

Recently, we proposed (particles of) montmorillonite (MMT) or MMT-rich bentonite clay as a low-cost and readily available adsorbent for biogas upgrading.^51^ MMT is a natural layered material of which the sorption capacity for nonpolar molecules like CO_2_ and CH_4_ can be tuned by varying the height of its interlayer galleries. This is achieved by exchanging its natural interlayer cations for others of different sizes. Cs^+^ is particularly suitable to achieve high sorption capacities for CO_2_ while excluding the larger CH_4_ molecules from the interlayer galleries.^51−53^ Consequently, Cs-bentonite demonstrates an equilibrium CO_2_/CH_4_ selectivity under near-ambient conditions of up to ∼35 (here defined as the ratio of [single-component] equilibrium CO_2_ sorption to equilibrium CH_4_ sorption at equal pressure and temperature).^51^ Importantly, it shows favorable regeneration within several minutes only under ambient-temperature N_2_ purge or vacuum conditions^51^ due to the only relatively weak adsorption of CO_2_ (|Δ*H*|≈30 kJ mol^–1^). Together, the rather high CO_2_/CH_4_ selectivity, the fast CO_2_ adsorption and desorption kinetics,^51^ and the low cost and high availability of the bentonite material suggest that Cs-bentonite could be an appealing alternative adsorbent for biogas upgrading.

However, the actual performance of an adsorbent in a vacuum pressure swing adsorption (VPSA) unit also depends crucially on the VPSA cycle configuration and the operating conditions (e.g., ambient temperature, feed biogas composition) and operating parameters (e.g., pressure, flow rate, step duration). Ultimately, the key process performance indicators are minimal specific energy consumption (here: energy use per amount of CH_4_ recovered in the output bio-CH_4_ product) and maximal productivity (here: the amount of feed biogas treated per kg sorbent per hour) while the specific requirements on product purity and component recovery are satisfied. (The product purity is the mole fraction of the desired component in that output product. The component recovery is the mole fraction of the component feed recovered in the appropriate output product.) Given the large number of possible VPSA cycle configurations, a range of possible operating conditions and parameters, and requirements on product purity and component recovery that differ per purpose (e.g., grid injection, liquefaction, chemical production) and between countries,^2,3,5^ this is most easily assessed numerically.

This work aims to (i) perform the numerical assessment of Cs-bentonite for biogas upgrading in a VPSA process, (ii) identify the optimal VPSA cycle configuration and operating parameters under the specific requirements on product purity and component recovery, and (iii) compare the performance of Cs-bentonite against conventional sorbents. We specifically focus on the production of bio-CH_4_ with a purity ≥0.90 that is compatible with grid injection in, e.g., The Netherlands.^2,3^ Simultaneously, in the light of (possibly forthcoming) strict regulations on CH_4_ emissions and the possibility to utilize a high-purity CO_2_ product, we also focus on high CH_4_ recovery ≥0.95 (i.e., high compared to earlier works, Section 3.5 and Table S1). We limit this benchmarking study to a small and simple unit with a target productivity of several Nm^3^ h^–1^ (normal cubic meters per hour) input biogas. This unit features a single upgrading stage and up to three columns. Hereon, we test seven different basic cycle configurations. While more complex cycle configurations,^7,38,54^ multiple units or upgrading stages (possibly hybrid, featuring different adsorbents or even different methods),^2,3,9,44,45,55^ auxiliary equalization tanks,^23,37^ and/or layered beds^30^ may achieve higher purity, recovery, and/or productivity, this is beyond the scope of the current work. We also consider the effects of the operating conditions, ambient temperature and feed biogas composition (i.e., CO_2_ fraction), on the process performance. The assessment makes use of a newly developed model that is validated against the experimental breakthrough and regeneration curves published in ref (51). Our numerical results confirm the excellent performance of Cs-bentonite for biogas upgrading and show particularly low specific energy consumption compared to conventional adsorbents.

## 2 Theoretical Basis

### 2.1 Model Assumptions

Various mathematical models to analyze biogas upgrading using a range of adsorbents were used in previous works. All these models had different levels of complexity and underlying assumptions, see, e.g., refs (56 and 57). In this article, we assess the VPSA process by using a newly developed nonisothermal and nonisobaric model. Herein, the following is assumed.

- The gas flow is represented by an axially dispersed plug flow model.
- The gas phases are described by the ideal gas law, *P* = ∑_*i*_*C*_g,*i*_*RT*.
- The mass transfer between the gas phases in the column void and the particle pore space and between the gas phase in the particle pore space and the adsorbed phase is described by linear driving force (LDF) models.
- The pressure gradient along the column is described by the Ergun equation.^58^
- Thermal equilibrium between the solid, gas, and adsorbed phases is established instantaneously. The local temperature of the solid, gas, and adsorbed phases is equal and described by a single energy balance.
- Heat exchange with the column wall occurs. The temperature of the column wall is constant and equal to the ambient temperature *T*_0_.
- There are no concentration, pressure, and temperature gradients in the radial direction of the column; i.e., the model is 1-dimensional (axial; *z*) in space along the length of the column.
- The properties of the column are uniform. The adsorbent particles are homogeneous, spherically symmetric, and equally sized.
- The input biogas is completely dry and devoid of minor impurities like H_2_S.

### 2.2 Model

**Figure 1:**
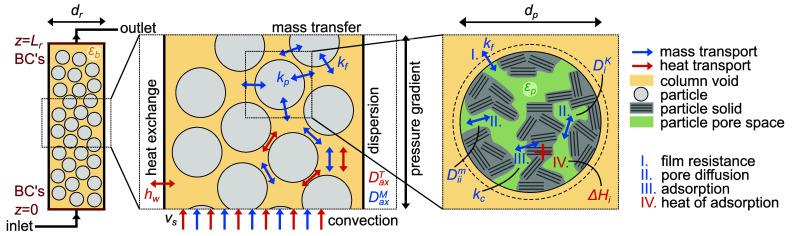
Schematic illustration of transport phenomena, coefficients, and properties on/in the (left) column scale, (center) column cross section, and (right) particle scale. (Details in the main text.)

The assumptions above result in mass balances for each of the different components in the gas phases and in the adsorbed phase, a momentum balance in the gas phase in the column void, and a combined energy balance for the solid, gas, and adsorbed phases. Figure 1 provides a schematic overview of the transport phenomena and coefficients and column and particle properties that are described and used in the model. The physical properties of the gas components (Table S2) and relations for the physical properties of the gas mixture (eqs S1–S6) and the transport coefficients (eqs S7–S14) are taken from the literature and discussed in the Supporting Information.

#### 2.2.1 Mass Balance (Column Void)

The mass balances for each component (with *i* ∈ {CO_2_, CH_4_, N_2_}) in the gas phase in the column void include convection, axial dispersion, and mass transfer between the gas phases in the column void and in the particle pore space, eq 1.
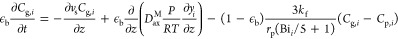
1Here, *C*_g,*i*_ is the concentration in the column void, *C*_p,*i*_ is the average concentration in the particle pore space, and *y*_*i*_ is the mole fraction in the gas phase in the column void, all of component *i*. ϵ_b_ is the column void fraction, *P* is the local pressure in the column void, *T* is the local temperature, *v*_s_ is the superficial gas velocity, and *D*_ax_^M^ is the axial mass dispersion coefficient. (The superficial gas velocity is the volumetric flow rate divided by the cross-sectional area of the column, without particles.) *k*_f_ is the film LDF coefficient, *r*_p_ is the particle radius, and Bi_*i*_ is the mass Biot number of component *i* (see below). The product 3*k*_f_/[*r*_p_(Bi_*i*_/5 + 1)] describes film resistance and pore diffusivity in series (see below). Substitution of the ideal gas law, *P* = ∑_*i*_*C*_g,*i*_*RT*, into eq 1 yields eq 2.
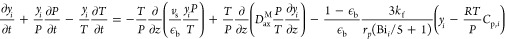
2

#### 2.2.2 Mass Balance (Particle)

The mass balances for each component in the particle pore space include adsorption and mass transfer to and from the gas phase in the column void, eq 3.
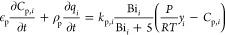
3Here, ϵ_p_ and ρ_p_ are the particle porosity and density, respectively, *q*_*i*_ is the particle-averaged adsorption, and *k*_p,*i*_ is the pore LDF coefficient from Glueckauf’s approximation,^59^eq 4, both of component *i*.
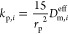
4Here, *D*_m,*i*_^eff^ is the effective pore diffusion coefficient of component *i* (which includes molecular and Knudsen diffusion; see Supporting Information, eq S10). Equation 4 is valid for *D*_m,*i*_^eff^*t*/*r*_p_^2^ > 0.1.^56^ Note that for Bi_*i*_ = *k*_f_*r*_p_/*D*_m,*i*_^eff^, eq 5 can be written:
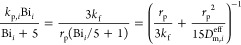
5Thus, the model describes film resistance and pore diffusion in series and mass is conserved between eqs 2 and 3. The rate of adsorption (i.e., mass transfer between the particle pore space and the adsorbed phase) is also described by an LDF model, eq 6.
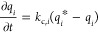
6Here, *q*_i_^*^ is the equilibrium adsorption of component *i* at temperature *T* and average concentration of component *i* in the particle pore space *C*_p,*i*_, as provided by the adsorption isotherms described in Section 2.3. *k*_c,*i*_ is the adsorption LDF coefficient of component *i*.

#### 2.2.3 Momentum Balance

The addition of the component mass balances in eq 2 under the condition ∑_*i*_*y*_*i*_ = 1 and the substitution of eq 3 therein yield the total mass balance, eq 7.
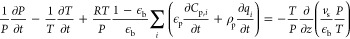
7The axial pressure gradient is related to the local superficial velocity by the semiempirical Ergun equation, eq 8.^58^
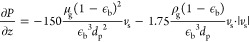
8Here, μ_g_ and ρ_g_ are the local viscosity and density of the gas, respectively, and *d*_p_ is the particle diameter. Equation 8 can be solved to find the local superficial velocity for a given pressure gradient. Alternatively, eq 8 provides a pressure gradient for a given (input) superficial velocity.

#### 2.2.4 Energy Balance

The energy balance accounts for heat effects due to adsorption and desorption, dispersive and convective energy transport via the gas phase in the column void, heat exchange between the gas phase in the column void and the column wall, and mass transfer between the gas phases and the adsorbed phase, eq 9.
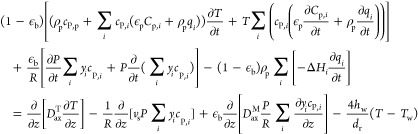
9Here, *c*_P,p_ and *c*_P,*i*_ are the heat capacities of the solid phase and of component *i* (assumed to be equal for the gas phases [in the column void and particle pore space] and the adsorbed phase), Δ*H*_*i*_ is the enthalpy of adsorption of component *i* (assumed to be independent of loading), *D*_ax_^T^ is the axial heat dispersion coefficient, *h*_w_ is the wall heat transfer coefficient, *d*_r_ is the column diameter, and *T*_w_ = *T*_0_ is the wall temperature set equal to the ambient temperature.

#### 2.2.5 Initial and Boundary Conditions

**Figure 2:**
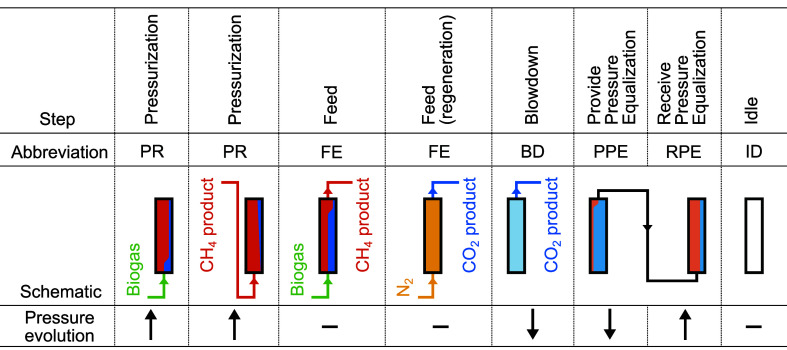
Steps used in the VPSA cycles. All schematics show the steps executed in the “forward” direction. The colors inside the column illustrate the typical component concentration in the column voids at the end of the respective step. Red: CH_4_; blue: CO_2_; yellow: N_2_; deeper colors indicate higher pressures. The N_2_ regeneration feed is only used for model validation, Section 2.4.

The system of partial differential equations (PDEs) discussed above requires suitable initial and boundary conditions. These depend on the specific steps in a VPSA cycle (configuration) that is defined by the order and direction in which these steps are executed. Figure 2 provides an overview of the specific steps that are used in this work and includes the abbreviations used to refer to these steps. The initial condition of the first step in the first simulated cycle is described by the absence of CO_2_ and CH_4_ in the column; instead, the column is saturated with N_2_ (assumed to be nonadsorbing) at the target regeneration pressure and at the ambient temperature. For each subsequent step, the final condition of the preceding step is used as an initial condition.

Table 1 displays the boundary conditions for the specific steps when executed in the “forward” direction. Analogous boundary conditions with both ends interchanged apply to steps that are executed in the “backward” direction. In summary, when an inlet (*z* = 0) or outlet (*z* = *L*_r_) is open, Danckwerts boundary conditions^56,60^ apply to the component mole fraction and the temperature. Specification of either the pressure or the gas velocity (effectively, the pressure gradient via eq 8) is required on both ends of the column. For a closed in- or outlet, *v*_s_ = 0 via the vanishing pressure gradient. The PR, FE, BD, and PPE steps include check valves that permit only a positive or zero inflow or outflow gas velocity (i.e., a negative or zero pressure gradient) using the min and max functions. *y*_in,*i*_ and *T*_in_ are the mole fraction of component *i* and the temperature, respectively, of the inlet gas stream. *P*_s,0_ and *t*_s,0_ are the local pressure and the time at the beginning of the step, respectively. *P*_PR_, *P*_BD_, τ_PR_, and τ_BD_ are the characteristic pressures and time scales within the respective steps.

**Table 1:** Boundary Conditions for Each Step Executed in the “Forward” Direction

step^a^	boundary conditions, *z* = 0	boundary conditions, *z* = *L*_r_
	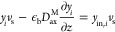	
pressurization (PR) →_0_⊂⊃_*L*_×	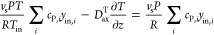	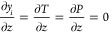
	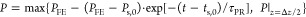	
	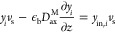	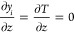
feed (FE) →_0_⊂⊃_*L*_→	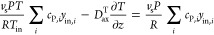	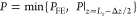
	Equation 8	
blowdown (BD) ×_0_⊂⊃_*L*_→	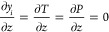	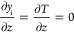
		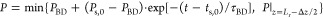
provide pressure equalization (PPE) ×_0_⊂⊃_*L*_→	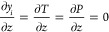	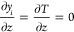
		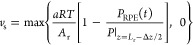
	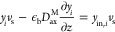	
receive pressure equalization (RPE) →_0_⊂⊃_*L*_×	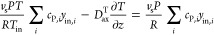	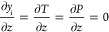
	Equation 8^b^	
idle (ID) ×_0_⊂⊃_*L*_↔	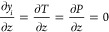	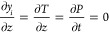

a Each step is illustrated schematically in Figure 2.

b While this boundary condition does not permit the specification of a molar flow rate, the combination of *v*_s_ and *P*(*v*_s_) that is consistent with the molar outflow rate of the PPE step is solved iteratively.

Some of the steps are coupled. First, the PPE and RPE require the conservation of mass and energy. The molar outflow rate during the PPE step is set proportional to the time-dependent pressure difference Δ*P*(*t*) between the outlet of that column and the inlet of the column in the RPE step. To calculate this pressure difference, the time evolution of the inlet pressure of the column in the RPE step in the preceding cycle is used. The molar outflow rate then is *v*_s_*A*_r_*P*/(*RT*) = max{0, *a*Δ*P*(*t*)}. Here, the coefficient *a* is analogous to a flow coefficient of a restricting device (we set *a* = 10^–5^ mol Pa^–1^ s^–1^). The output (i.e., the time-dependent molar flow rate, composition, and temperature) of the PPE step is then used as an input for the RPE step thereafter. This allows for multiple columns to be simulated iteratively by using a single column, instead of simultaneously. This (“store-and-retrieve”) method is known to introduce oscillations around the equalization pressure.^61^ However, in our simulations, these oscillations decay well before the final cycle is simulated. Second, (part of) the output product of a preceding step (i.e., feed or blowdown) can serve as an input for another step (i.e., pressurization or purge/rinse feed; the latter is not used in this work). In this case, the average composition of the output product is taken and recycled at the ambient temperature and pressure.

When a step needs to be simulated before the step from which it requires input has been simulated for the first time, an initial estimate is used. For the simulation of the PPE step(s), a constant pressure between the target feed and blowdown pressures is taken as the inlet pressure of the RPE step(s). In the case of recycled products, the composition of the biogas is taken instead. For some representative cases, we verified that the cyclic steady state is independent of these initial estimates.

#### 2.2.6 Model Implementation

To solve the system of PDEs, the column was divided into *N* = 30 cells, each with a length Δ*z*. The PDEs were spatially discretized using the weighted essentially non oscillatory (WENO)-finite volume method,^62^ as is detailed in the Supporting Information (eqs S15–S19). The discretized system of coupled ordinary differential equations was solved by using the ode15s solver in MATLAB (R2023b) with an absolute tolerance of 10^–3^. The solution was refined 10 times for higher accuracy of the Riemann-type integration for the in- and output flows. To ensure that the cyclic steady state was reached, 100 cycles were simulated in each simulation. The typical computation time of one simulation was around 1 h on a dedicated desktop PC with one Intel i7–7700 CPU (3.60 GHz) and 8.00 GB of RAM.

### 2.3 Separation System

**Figure 3:**
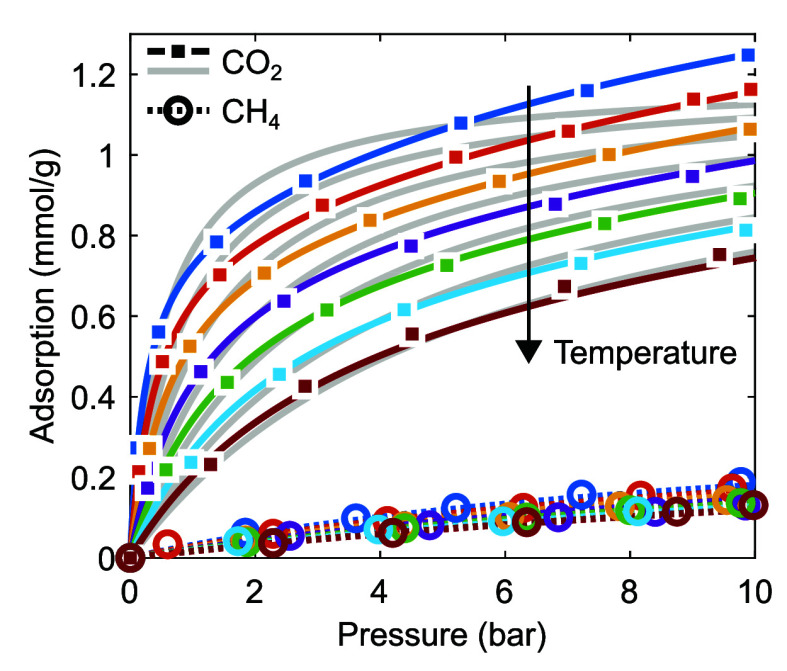
Adsorption isotherms of CO_2_ and CH_4_ on binderless Cs-bentonite. For CO_2_, the arrow indicates increasing temperature from 10 to 70 °C at 10 °C increments. For CO_2_ and CH_4_, identical colors indicate equal temperatures. The experimental data is presented with solid squares (CO_2_) and open circles (CH_4_). The temperature-dependent fits are presented with colored solid lines (CO_2_; DSL), gray solid lines (CO_2_; SSL), and colored dashed lines (CH_4_; SSL).

The experimental adsorption isotherms of CO_2_ and CH_4_ on Cs-bentonite are presented and discussed in ref (51). Therein, each isotherm was fitted individually with a (single- or dual-site) Langmuir model (SSL and DSL). As the simulations require the isotherms at intermediate temperatures, the data is refitted with a temperature-dependent model, eq 10.
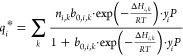
10Here, the fit parameters are *n*_*i*,*k*_ the number of adsorption sites per unit sorbent mass of type *k* for gas component *i* and *b*_0,*i*,*k*_ and Δ*H*_*i,k*_ the ratio between the adsorption and desorption attempt frequency and the enthalpy of adsorption for sites of type *k* for gas component *i*, respectively, and *y*_*i*_ = 1. We fit both sets of adsorption isotherms with the SSL model (i.e., *k* = 1) and also fit a DSL model (i.e., *k* ∈ {1,2}) to the CO_2_ adsorption isotherms only, in line with ref (51). The fitted adsorption isotherms along with the experimental data in the temperature range of 10–70 °C and the corresponding fit parameters are presented in Figure 3 and Tables S3 and S4, respectively.

The CO_2_ adsorption is described much better by the DSL model than by the SSL model—in particular at lower temperatures—due to the larger number of fitting parameters. We use the fitted DSL model to describe the equilibrium CO_2_ adsorption *q*_i_^*^ in eq 6. In contrast, we use the Δ*H*_*i*,1_ fitted using the SSL model as an effective enthalpy of adsorption for the energy balance, eq 9. For CH_4_, the fitted SSL model is used for both the equilibrium adsorption and the energy balance. We further multiply the equilibrium adsorption by 0.91 to account for the (assumed passive) binder material in a mass fraction of 0.09 required to produce particles from the binderless Cs-bentonite in Figure 3.^51^

The synergistic and competitive adsorption of multiple components on MMTs is a complex process that cannot easily be described by competitive adsorption models. This is due to the violation of various assumptions made in such models. Most notably, the adsorption sites are not homogeneous and the number of available adsorption sites differs per component due to steric limitations—and may in fact increase (due to swelling) or decrease by the coadsorption of another component.^51^ In this previous work, we noted that these effects are minor for the coadsorption of CO_2_ and CH_4_ on Cs-bentonite. Hence, we choose not to include these minor effects in the model (i.e., the adsorption of the different gas components occurs independently).

### 2.4 Model Validation

**Figure 4:**
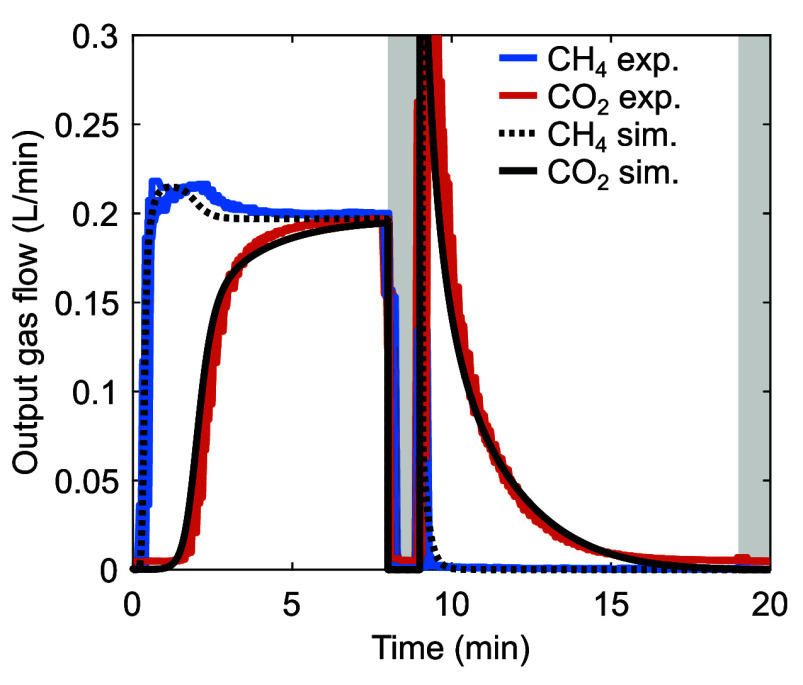
Comparison between experimental^51^ and simulated cyclic breakthrough and regeneration measurements on 50 g of Cs-bentonite particles, *T*_0_ ≈ 23 °C. Column dimensions: *d*_r_ = 1.3 cm and *L*_r_ = 60 cm. Each cycle consisted of (i) a 8 min forward feed step with an inlet gas mixture containing only CO_2_ (mole fraction of 0.50 ± 0.02) and CH_4_ (mole fraction of 0.50 ± 0.02) at a total flow rate of 0.40 L min^–1^, (ii) a 1 min forward idle step (gray background), (iii) a 10 min forward feed step with N_2_ (for regeneration) at a flow rate of 0.60 L min^–1^, and (iv) a 1 min forward idle step (gray background). The volumetric flow rates are actual values. The experimental cycles are shifted on top of each other. The estimated material properties are tabulated in Table 2.

To validate the model and to estimate *k*_c_ and *d*_m_ (i.e., the typical pore diameter; an input parameter for *D*_m,*i*_^eff^), we compare the output flow compositions of experimental consecutive (cyclic) breakthrough and regeneration (in N_2_) measurements^51^ with simulated output flow compositions under the same conditions, Figure 4. Table 2 lists the estimated material properties.

**Table 2:** Material Properties of Cs-Bentonite Particles^a^

*d*_m_ (nm)	15
*k*_c_ (s^–1^)^b^	0.10
*d*_p_ (mm)	3.0
ρ_p_ (kg m^–3^)	1400
ρ_c_ (kg m^–3^)^c^	2700^63,64^
ϵ_p_ (−)	0.48
τ(−)^d^	2.0
*c*_P,p_(kJ kg^–1^ K^–1^)	0.80^65^

a *d*_m_ and *k*_c_ are estimated from the experimental breakthrough curves, while all other properties are estimated from the literature or macroscopic properties of the materials.

b Set equal for CO_2_ and CH_4_ and independent of temperature.

c Crystal density.

d Particle tortuosity.

The simulated outputs can describe the experimental outputs well, both during the feed step with the mixture of CO_2_ and CH_4_ and during the regeneration step using N_2_ feed. To cross-validate the estimated values for *d*_m_ (that ultimately affects the diffusional transport in particles) and *k*_c_ (that sets the “intrinsic” adsorption kinetics, i.e., into the interlayer galleries), we compare the corresponding LDF coefficients with independent kinetic experiments of CO_2_ adsorption on powders and small and large particles of Cs-bentonite, Figure 4 in ref (51). As for the diffusional transport limitations, for CO_2_ and at 20 °C, typically Bi_CO_2__≫1 and the molecular and Knudsen diffusivities of CO_2_ are on the order of 1.6 × 10^–5^ m^2^ s^–1^ and 1.9 × 10^–6^ m^2^ s^–1^, respectively (see Supporting Information, eqs S7 and S11). This indicates that mass transfer between the gas phase in the column void and the particle pore space is mostly limited by Knudsen diffusion in the particle pore space. For ϵ_p_ = 0.48 and τ = 2.0, the product , i.e., approximately the inverse of the pore LDF coefficient, yields typical time scales of 0.7 and 18 s for small particles with *r*_p_ = 2 mm and large particles with *r*_p_ = 1 cm, respectively. These are in line with the previous observations that for small particles, the CO_2_ adsorption nearly follows the adsorption on a powder, and for large particles, the CO_2_ adsorption follows the adsorption on a powder within ∼10–20 s. Moreover, the estimated effective pore diameter *d*_m_ = 15 nm is close to the typical mesopore throat size and between the typical micro- and mesopore body sizes of MMT^66^ (see also Figure 4.10 in ref (67)). As for the “intrinsic” adsorption, *k*_c_^–1^ = 10 s agrees well with the fast CO_2_ adsorption and desorption on powders (and small particles) that follows the CO_2_ concentration of the environment within ∼10 s. Ultimately, these independent kinetic experiments thus confirm the (order of magnitude) of our estimated values for *d*_m_ and *k*_c_.

### 2.5 Process Definition

#### 2.5.1 VPSA Cycle Configurations

We consider seven different cycle configurations that are also illustrated schematically in Figure S1.

1. (i) Forward pressurization with biogas (PR); (ii) forward feed with biogas (FE; CH_4_ product collection); and (iii) backward blowdown (BD; CO_2_ product collection).
2. As configuration 1 but (i) backward pressurization with the CH_4_ product (PR).
3. (i) Forward pressurization with biogas (PR); (ii) forward feed with biogas (FE; CH_4_ product collection); (iii) forward provide pressure equalization (PPE); (iv) backward blowdown (BD; CO_2_ product collection); and (v) forward receive pressure equalization (RPE).
4. As configuration 3 but (v) backward receive pressure equalization (RPE).
5. As configuration 3 but (i) backward pressurization with the CH_4_ product (PR).
6. As configuration 3 but (i) backward pressurization with the CH_4_ product (PR) and (v) backward receive pressure equalization (RPE).
7. (i) Forward pressurization with biogas (PR); (ii) forward feed with biogas (FE; CH_4_ product collection); (iii) forward provide pressure equalization (PPE); (iv) backward provide pressure equalization (PPE); (v) backward blowdown (BD; CO_2_ product collection); (vi) forward receive pressure equalization (RPE); (vii) idle (ID; required for column synchronization); and (viii) backward receive pressure equalization (RPE).

Configuration 1 is the minimal configuration for a VPSA process. For configurations 2 and 5–6, the pressurization with the CH_4_ product (instead of biogas) possibly increases the CH_4_ purity.^41,43^ This is, to a certain extent, similar to the often applied CH_4_ purge step that improves the removal of CO_2_ from the column in particular at the end where the CH_4_ product is collected.^8,10,22,23,25,26,36,37,39−41,45,46^ We do not consider product purge/rinse steps in the current work. For configurations 3–7, the pressure equalization steps possibly increase the CH_4_ recovery and decrease the specific energy consumption.^6,7,10,22,24,25,34,37−40,44−46,61,68,69^ These pressure equalization steps require two columns to be connected at specific times and, thereby, the synchronization of multiple columns. The synchronization scheme for two (one PE step; configurations 3–6) or three (two PE steps; configuration 7) columns is given in Table 3.

**Table 3:** Column Synchronization Scheme

column	configurations 1–2		
1	PR	FE	BD

column	configurations 3–6					
1	PR	FE	PPE	BD	BD	RPE
2	BD	BD	RPE	PR	FE	PPE

column	configuration 7								
1	PR	FE	PPE	PPE	BD	BD	RPE	ID	RPE
2	RPE	ID	RPE	PR	FE	PPE	PPE	BD	BD
3	PPE	BD	BD	RPE	ID	RPE	PR	FE	PPE

#### 2.5.2 Operating Parameters

The process operating parameters include the pressure (evolution) during, duration of, and inflow rate during each step. Four of these operating parameters, namely, the (target) pressurization pressure *P*_PR_ = 1–4 bar, the (target) blowdown pressure *P*_BD_ = 0.05–0.15 bar, *Q*_in_ = 0.3–1.0 (see below), and the feed duration *t*_FE_ = 1–10 min, are varied in our simulations within the indicated ranges. *Q*_in_ is a fraction that relates the CO_2_ inflow during the feed step to the estimated equilibrium CO_2_ working capacity of the sorbent, . Then, the inlet superficial velocity . Here, *m*_s_ is the sorbent mass in the column,  is the CO_2_ mole fraction in the biogas, and *A*_r_ is the cross-sectional area of the column. By increasing *Q*_in_, the column utilization thus increases and the (expected) position of the CO_2_ breakthrough front at the end of the FE step shifts to (or beyond) the outlet end of the column. Moreover, this position is (nearly) independent of *P*_PR_, *P*_BD_, and *t*_FE_ for a fixed *Q*_in_. However, *Q*_in_ does not take into account the CO_2_ that is provided during any other step between BD and FE.

The other operating parameters are either set constant or depend only on the previously discussed operating parameters. These are (i) the duration of the PR, PPE, and RPE steps that are fixed at 1 min, (ii) the duration of the BD step that is set equal to the combined duration of the PR and FE steps for column synchronization (Table 3), and (iii) the FE pressure that is set equal to the (target) PR pressure. Furthermore, the characteristic pressurization and blowdown times for the exponential pressure in- or decrease, τ_PR_ and τ_BD_, are set to 1/5th of the duration of the respective steps. At the end of these steps, the target pressure is then achieved within around 99.5%.

#### 2.5.3 Performance Indicators

The purity and recovery of product and component *i* are calculated from the cumulative molar output *n*_*i*,out_ and input *n*_*i*,in_ of component *i* over all steps in one cycle in the cyclic steady state. Superscripts product_*i*_ and BG indicate whether the out/input is directed to/from product *i* or from biogas (see Section 2.5.1). The recovery of component *i* is expressed as the ratio between the recovered component in output product *i* (after accounting for any recycled stream) and the input of that component, eq 11.
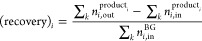
11Here, *k* sums over all steps in one cycle in the cyclic steady state. The purity of product *i* is expressed as the ratio between the desirably collected component *i* and the total collected product, eq 12.
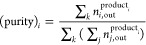
12Here, *k* sums over all steps in one cycle in the cyclic steady state and *j* sums over all gas components. The compression and evacuation processes are approximated to be adiabatic processes, eq 13. The required power *p* is calculated under the assumption that the feed gas pressure and the vacuum pump discharge pressure are equal to the ambient pressure *P*_0_.
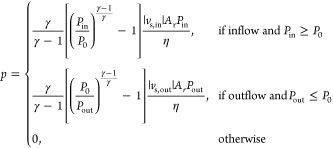
13Here, γ is the adiabatic constant (we assume γ = 1.3) and η the mechanical efficiency (we assume η = 0.8). *P*_in_ and *v*_s,in_ and *P*_out_ and *v*_s,out_ are the inlet and outlet pressure and superficial velocity, respectively. Compression of the product(s) beyond ambient pressure is not considered. The PPE and RPE step pair, any idle step, pressurization and feed to/at (sub)atmospheric pressure, and blowdown to(ward) atmospheric pressure do not require power. The specific energy consumption (SEC) is defined as the energy consumption per Nm^3^ (normal cubic meter; i.e., at 15 °C and 1 atm) collected CH_4_ in the CH_4_ product, eq 14.
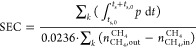
14Here, *t*_s_ is the duration of the step. The productivity is defined as the amount of input biogas (in Nm^3^) per kg adsorbent per hour, eq 15.
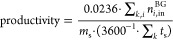
15

#### 2.5.4 Column Sizing

To match (within the order of magnitude) the capacity of a small “farm-scale” unit, we simulate a column with *L*_r_ = 2.0 m and *d*_r_ = 20 cm, loaded with 55 kg of Cs-bentonite particles (ϵ_b_ = 0.375). For reference, the CO_2_ adsorption capacity of the column at the typical temperature of 15 °C and a CO_2_ partial pressure of 0.45 bar is around 27.5 mol or 0.65 Nm^3^ CO_2_.

## 3 Results and Discussion

### 3.1 VPSA Cycle

**Figure 5:**
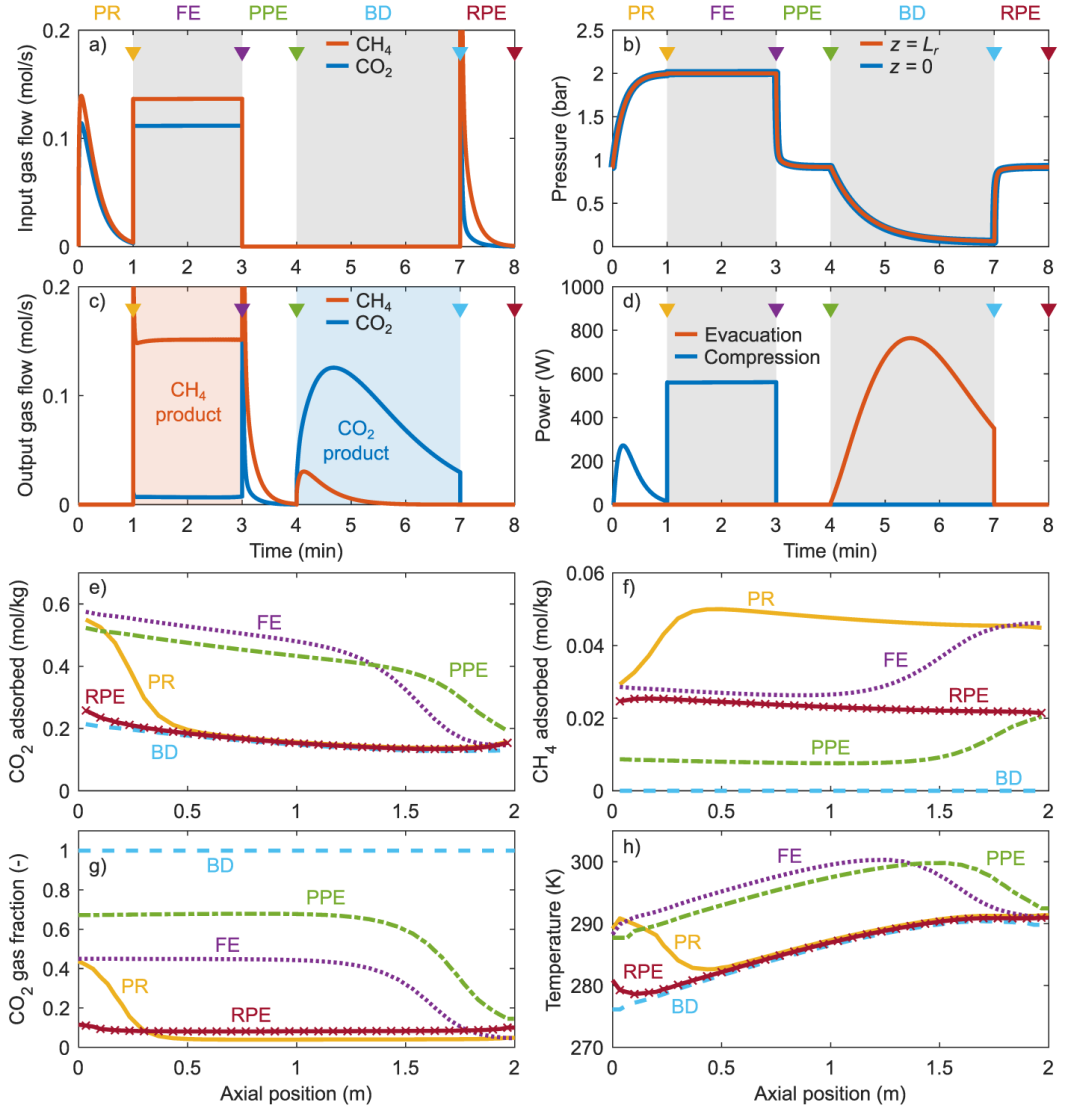
Simulation results (CSS) of configuration 3, , *T*_0_ = 15 °C, and operating parameters *P*_PR_ = 2 bar, *P*_BD_ = 0.05 bar, *t*_FE_ = 2 min, and *Q*_in_ = 0.5. (a) Input gas flows, (b) pressure, (c) output gas flows, and (d) power consumption over time. (Steps are indicated by the alternating background colors.) (e–h) Profiles along the column axial dimension at the end of each step, of (e) CO_2_ adsorbed, (f) CH_4_ adsorbed, (g) CO_2_ mole fraction in the column void, and (h) temperature. Line colors in (e–h) correspond to the color of the downward triangles in (a–d).

To illustrate the principle of the VPSA process, Figure 5 shows the results of one representative simulation using configuration 3 at an ambient temperature of 15 °C and with biogas feed mole fractions of 0.45 CO_2_ and 0.55 CH_4_. Specifically, Figure 5a–d show the input gas flow, pressure, output gas flow, and power consumption, respectively, during the final five simulated cycles (i.e., cycles 96–100). Figure 5e–h show the adsorbed CO_2_, adsorbed CH_4_, CO_2_ mole fraction in the column void, and temperature, respectively, along the axial dimension and at the end of each step (corresponding to the downward triangles in Figure 5a–d) during the final five simulated cycles. As all these results overlap between each of the final five simulated cycles, we conclude that the cyclic steady state was achieved.

First, during the PR step, biogas is provided at *z* = 0 while the other end of the column is closed. This increases the column pressure until the desired *P*_PR_ is (nearly) reached. The CO_2_ in the biogas is mostly adsorbed near the column inlet. As a consequence, also the temperature and CO_2_ gas fraction increase only near the column inlet. In turn, the adsorbed CH_4_ and CH_4_ gas fraction increase in the center and toward the opposite end of the column.

Second, during the FE step, biogas is provided at *z* = 0 and the CH_4_ product is collected at *z* = *L*_r_. The column pressure remains nearly constant, besides the (slightly varying) pressure drop over the column that is of the order ∼10 mbar. The CO_2_ adsorption front and the temperature front progress synchronously along the axial dimension. The FE step is stopped before the CO_2_ adsorption front reaches the column outlet. Meanwhile, the FE step also reduces the amount of adsorbed and gaseous CH_4_ in the column. However, the amount of CO_2_ that is collected in the CH_4_ product, while much smaller than in the biogas, is still significant due to the incomplete regeneration of the sorbent during the BD step (see below).

Third, during the PPE step, the column is connected at *z* = *L*_r_ to another column that was previously evacuated in a BD step and is now in the RPE step (Table 3). The pressure difference between both columns facilitates the mass transfer from this column to the column in the RPE step until a pressure equilibrium is reached. The gas flow is relatively rich in CH_4_. As this CH_4_ is now not collected in the CO_2_ product (in the BD step hereafter), this significantly increases the CH_4_ recovery. In contrast, most of the (adsorbed) CO_2_ is retained in the column.

Fourth, during the BD step, the CO_2_ product is collected by backward evacuation at *z* = 0 while the other end of the column is closed. This decreases the column pressure until the desired *P*_BD_ is (nearly) reached. The lower pressure reduces the adsorbed CO_2_ to ∼0.2 mol kg^–1^ and thereby results in a decrease of the temperature. Essentially no CH_4_ is retained in the column at the end of the BD step, i.e., the CO_2_ gas fraction is near unity. Nevertheless, the amount of CH_4_ that is collected in the CO_2_ product is small as most CH_4_ was removed from the column in the preceding PPE step.

Fifth, during the RPE step, the input gas is provided at *z* = 0 by another column that is in the PPE step until a pressure equilibrium is reached between both columns. The input gas that is relatively rich in CH_4_ (see above) increases the amount of adsorbed CH_4_ and decreases the CO_2_ gas fraction. The amount of adsorbed CO_2_ and the temperature remain nearly constant due to the only small amount of CO_2_ in the input gas. At this point, one cycle is completed and another is started with the PR step.

As for energy consumption, the PR and FE steps require input gas at above-ambient pressure and thus compression power. Similarly, the BD step is performed at subambient pressure and requires evacuation power. In contrast, the PPE and RPE steps require no power, as they are driven by an initial pressure difference between both columns.

The performance indicators are obtained by the integration of the input and output gas flows and the power consumption. For this specific configuration and this specific combination of operating parameters, the CH_4_ purity is 0.9568, the CH_4_ recovery is 0.9387, the SEC is , and the productivity is 0.1133 Nm_BG_^3^ kg^–1^ h^–1^. The performance indicators can possibly, however, be enhanced when a different combination of operating parameters is used. The effect of changing only one of the operating parameters while all others are fixed is discussed in the Supporting Information, Figure S2 and Table S5. These results suggest (i) a trade-off between performance indicators (e.g., CH_4_ purity versus CH_4_ recovery and SEC versus productivity) and (ii) interdependence of the (optimal) operating parameters. Thus, one-dimensional sensitivity analyses do likely not provide the optimal combination of operating parameters.

### 3.2 Effect of Cycle Configuration

The process performance depends crucially on the VPSA cycle configuration and the operating parameters and conditions. Maximizing the performance, therefore, requires (i) selecting for each configuration the combination of operating parameters that result in the maximum productivity and minimum SEC under the specific constraints that are set on output gas purity and recovery and based thereon (ii) identifying the optimal configuration, all under the given operating conditions. To this end, each configuration was first simulated with all element combinations of the operating parameter sets *P*_PR_ = {1,1.5,2,4} bar, *P*_BD_ = {0.05,0.10,0.15} bar, *t*_FE_ = {1,2,5,10} min, and *Q*_in_ = {0.4,0.5,0.6,0.65,0.7,0.8} and optionally *Q*_in_ = {0.3,0.9,1.0} (i.e., ≥288 combinations per configuration), again at an ambient temperature of 15 °C and with biogas feed mole fractions of 0.45 CO_2_ and 0.55 CH_4_. We refer to these combinations of operating parameters as the “seed”. Additional, more favorable combinations of operating parameters were selected from an interpolation of the output performance indicators of the initial seed simulations (and any other preceding generation of simulations) on a refined grid of operating parameters using the *griddatan* function in MATLAB. The selected combinations of operation parameters were then used as inputs for additional simulations. The interpolation method allows for a relatively rapid screening of combinations of operating parameters. This method is reasonably accurate, as demonstrated in the parity plots between the interpolated (based on the initial seed only) and additionally simulated (all simulations other than the initial seed) outputs in Figure S3.

**Figure 6:**
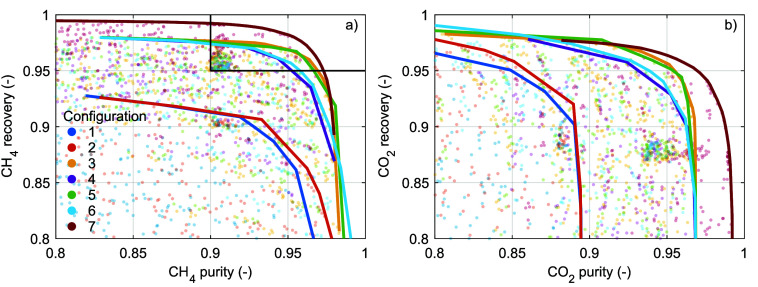
Simulated (opaque dots) and maximum (solid lines) component recovery as a function of component purity for (a) CH_4_ and (b) CO_2_. *T*_0_ = 15 °C; . The different colors indicate the different configurations (Section 2.5.1).

Figure 6a,b display, for all simulated combinations of operating parameters and for all configurations, the CH_4_ recovery as a function of the CH_4_ purity and the CO_2_ recovery as a function of the CO_2_ purity, respectively (opaque dots). Most of the simulated combinations result in CH_4_ recoveries and/or CH_4_ purities that do not satisfy our constraints (indicated by the top-right box in Figure 6a). The maximum component recovery as a function of the component purity is for each configuration indicated by the solid lines. For both CH_4_ and CO_2_, the maximum recovery decreases with increasing purity. This is in accordance with previous studies on various sorbent materials^8,10,14,20,22,24,26,32,37,39,40,42,45^ and with the trade-off between both performance indicators suggested by Figure S2 and Table S5. By comparing the different configurations, we conclude the following. First, the pressure equalization steps in configurations 3–7 significantly increase the maximum CH_4_ recovery and CO_2_ purity, as was also found in previous studies.^22,37,38^ This increase is due to the removal of CH_4_ from the column during the PPE step before the CO_2_ product is collected during the BD step. Second, the pressurization with the CH_4_ product in configurations 2 and 5–6 can improve the maximum CH_4_ purity and CO_2_ recovery. A (relatively pure) CH_4_ product then displaces the CO_2_ adsorption front from the column end where the CH_4_ product is collected. Third, the RPE step in the forward direction (configurations 3 and 5) can improve the combination CH_4_ recovery and CH_4_ purity and the combination CO_2_ recovery and CO_2_ purity as compared to the RPE step in backward direction (configurations 4 and 6) when the CH_4_ recovery is relatively high. In contrast, a RPE step in the backward direction can improve the maximum CH_4_ purity and CO_2_ recovery when the CH_4_ recovery is relatively low, similar to the pressurization with the CH_4_ product. The effect of the RPE direction is discussed further in the Supporting Information, Figure S4. For a minimal CH_4_ purity of 0.90, the maximum CH_4_ recovery is ∼0.913 for configurations 1–2, ∼0.975 for configurations 3–6, and ∼0.992 for configuration 7. In other words, CH_4_ recovery above ∼0.91 requires at least two connected columns (i.e., configurations 3–7). Similarly, CH_4_ recovery above ∼0.97 requires at least three connected columns (configuration 7).

**Figure 7:**
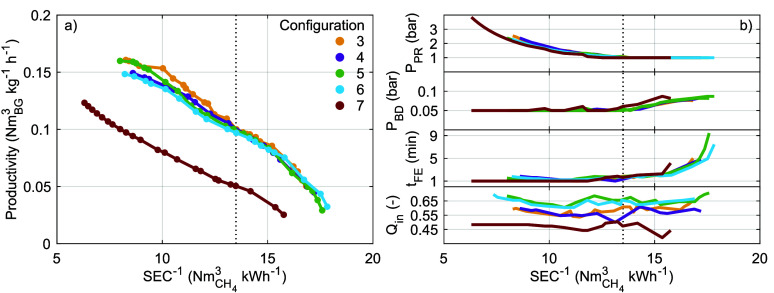
(a) Pareto fronts for the maximum productivity and minimal SEC under the constraints CH_4_ purity ≥0.90 and CH_4_ recovery ≥0.95. (b) Operating parameters along the Pareto fronts. *T*_0_ = 15 °C; . The different line colors indicate the different configurations (Section 2.5.1).

For each configuration, Figure 7a displays the Pareto front for the maximum productivity and minimum SEC (or maximum SEC^–1^), all under the constraints CH_4_ recovery ≥0.95 and CH_4_ purity ≥0.90 (top-right box in Figure 6a). As configurations 1–2 never satisfy these constraints, these do not show in this figure. The maximum productivity decreases with decreasing SEC. This is similar to the trade-off between product purity and recovery and was also suggested by Figure S2 and Table S5. Remarkably, configurations 3–6 show a nearly identical decrease of the maximum productivity with decreasing SEC. To understand this similarity, we compared the CO_2_ adsorption profiles (as in Figure 5e) specifically for simulations around the center of the Pareto front (). This revealed quite similar profiles between the different configurations at the end of the FE, PPE, and BD steps. Apparently, for the current, relatively mild constraint on CH_4_ purity ≥0.90, the pressurization gas and the direction of the RPE step do not significantly affect the process performance (see Supporting Information; Figure S4). Configuration 7 shows a lower productivity for a given SEC than configurations 3–6, despite its ability to achieve higher CH_4_ recovery. This reduced productivity can be attributed to the longer cycle duration due to the required “idle” step for column synchronization and to the presence of two PPE and RPE steps in this configuration, see also refs (37 and 69). Ultimately, these results imply that the VPSA cycle configuration should be tailored toward the specific requirements on product purity and component recovery and that the configuration that yields the highest product purity and/or component recovery does not necessarily provide the optimal process performance.

Figure 7b shows the operating parameters along the Pareto fronts. *P*_PR_, *P*_BD_, and *t*_FE_ do not depend strongly on the specific configuration but vary along the Pareto front. When high productivity is desired over low SEC (left side in Figure 7a–b), *P*_PR_ is high and *P*_BD_ and *t*_FE_ are at or close to their lower bounds of 0.05 bar and 1 min, respectively. With decreasing SEC and productivity, first *P*_PR_ decreases toward its lower bound of 1 bar, while *t*_FE_ increases only slightly and *P*_BD_ remains at its lower bound (up to the black dotted vertical line at ). Upon further decreasing the SEC at the expense of productivity (right side in Figure 7a–b), both *P*_BD_ and *t*_FE_ increase while *P*_PR_ remains at its lower bound of 1 bar. This indeed confirms that the operating parameters are interdependent and that each individual operating parameter should not be “optimized” using a one-dimensional sensitivity analysis only. In contrast to the other operating parameters, *Q*_in_ is relatively constant along the Pareto front but differs between configurations. Specifically, *Q*_in_ ≈ 0.56 for configurations 3–4, *Q*_in_ ≈ 0.65 for configurations 5–6, and *Q*_in_ ≈ 0.46 for configuration 7. Recall that *Q*_in_ reflects the fraction of the expected CO_2_ working capacity that is provided during the FE step only. The differences between the configurations can then be attributed to the amount of CO_2_ that is already provided during the PR and RPE steps. Compared to configurations 3–4 in which the PR step uses biogas, less CO_2_ is provided during the PR step in configurations 5–6 that use the CH_4_ product instead. This permits a larger *Q*_in_ in configurations 5–6 before CO_2_ contaminates the CH_4_ product. Compared to configurations 3–4 that use one RPE step, more CO_2_ is provided during the two RPE steps in configuration 7. This permits a smaller *Q*_in_ in configuration 7 before CO_2_ contaminates the CH_4_ product.

The effects of the individual operating parameters on the performance indicators are for all configurations illustrated in Figures S5–S9. We can now generalize these along all dimensions and for all configurations. In summary, (i) increasing *P*_PR_ increases the productivity and SEC and generally decreases the CH_4_ recovery while the effect on CH_4_ purity is nontrivial and depends on the other operating parameters; (ii) increasing *P*_BD_ decreases the CH_4_ recovery and productivity and almost always decreases the CH_4_ purity and the SEC (configurations 2 and [less so] 5–6 that use CH_4_ product pressurization deviate at high *P*_PR_ only); (iii) increasing *t*_FE_ increases the CH_4_ purity and decreases the productivity; and (iv) increasing *Q*_in_ increases the CH_4_ recovery at the expense of CH_4_ purity, increases the productivity, and decreases the SEC. These trends are also in accordance with those presented in Figure 7b for fixed constraints on CH_4_ recovery and purity and largely in line with the observations for configuration 3 along one dimension only (i.e., Figure S2 and Table S5).

### 3.3 Effects of Ambient Temperature and Biogas Composition

To illustrate the effects of the operating conditions ambient temperature and biogas composition on the process, we performed additional simulations with (i) *T*_0_ = 25 °C and  and (ii)  and *T*_0_ = 15 °C, in addition to (iii) the previously discussed conditions (*T*_0_ = 15 °C, ; all balance CH_4_). We restrict to configuration 3 for three main reasons. First, two connected columns (configurations 3–6) result in the highest productivity for a given SEC under the constraints CH_4_ purity ≥0.90 and CH_4_ recovery ≥0.95. Second, the RPE step in the forward direction (configurations 3 and 5) can improve the combination CH_4_ recovery and CH_4_ purity in the relevant domain as compared to the RPE step in the backward direction (configurations 4 and 6). Third, configuration 3 (and 4) excludes CH_4_ product refluxes and hence simplifies the unit (as compared to configurations 5–6).

**Figure 8:**
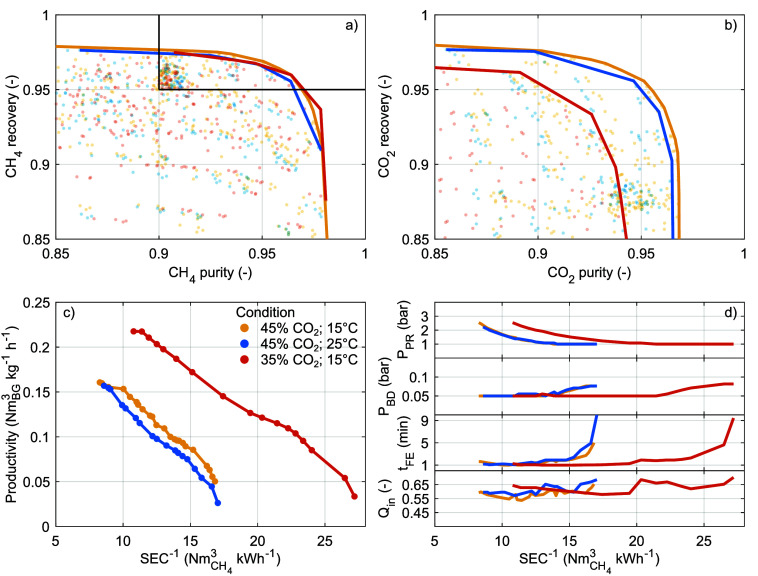
Simulated (opaque dots) and maximum (solid lines) component recovery as a function of component purity for (a) CH_4_ and (b) CO_2_. (c) Pareto fronts for the maximum productivity and minimal SEC under the constraints CH_4_ purity ≥0.90 and CH_4_ recovery ≥0.95. (d) Operating parameters along the Pareto fronts. Configuration 3; ambient and feed biogas conditions as detailed in legend.

Figure 8a,b display for each simulated combination of operating parameters and for the three conditions the component recovery as a function of the component purity (similar to Figure 6). The different temperature and biogas composition only have limited effects on the maximum CH_4_ recovery and CH_4_ purity (Figure 8a). Consequently, under all three conditions, the constraints CH_4_ purity ≥0.90 and CH_4_ recovery ≥0.95 can be satisfied. In contrast, but as expected, a smaller CO_2_ fraction in the biogas reduces the maximum CO_2_ recovery and CO_2_ product purity (Figure 8b).

Figure 8c displays the Pareto front for the maximum productivity and minimal SEC under the constraints CH_4_ recovery ≥0.95 and CH_4_ purity ≥0.90 (similar to Figure 7a). First, a higher ambient temperature slightly decreases the productivity and/or increases the SEC. This reduced performance can be attributed to the reduced CO_2_ adsorption at higher temperatures, Figure 3.^51^ This contrasts some other sorbents for which higher temperatures that facilitate their regeneration and/or enhance the diffusivity of CO_2_ therein are preferred.^22,24,36,42^ Second, a smaller CO_2_ fraction in the biogas strongly increases the productivity and/or decreases the SEC, in accordance with earlier studies.^6,8^ This improved performance can be attributed to the larger amount of CH_4_ that can be processed per unit adsorbed CO_2_. Figure 8d shows the operating parameters along the Pareto front. The trends herein largely resemble those for the different configurations (Figure 7b) but are shifted toward lower SEC for a smaller biogas CO_2_ fraction.

### 3.4 Alternative Requirements on Product Purity and Component Recovery

**Figure 9:**
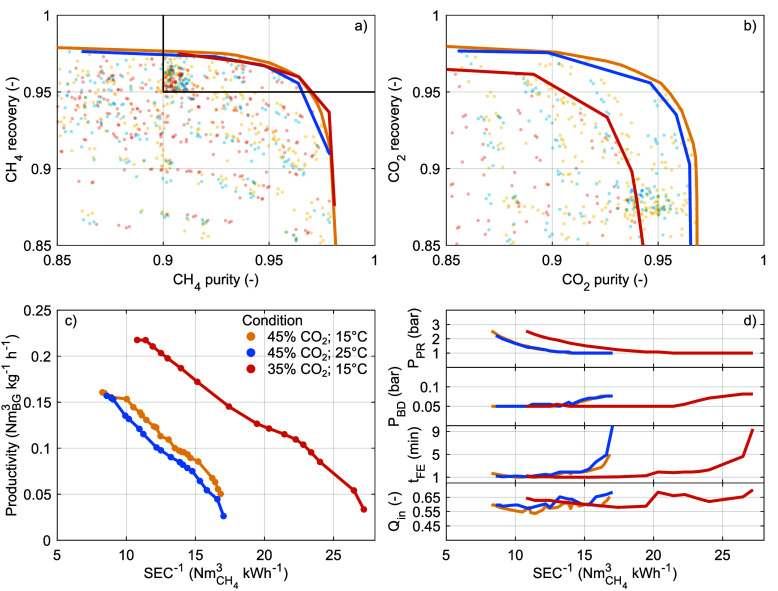
Interpolated Pareto fronts for the maximum productivity and minimal SEC under the constraints (a) CH_4_ recovery ≥0.90 and variable minimal CH_4_ purity and (d) CH_4_ purity ≥0.90 and variable minimal CH_4_ recovery. (b,c) Operating parameters along the Pareto fronts in (a). (e,f) Operating parameters along the Pareto fronts in (d). Configuration 3; *T*_0_ = 15 °C; . The arrows are detailed in the main text.

The requirements on product purity and component recovery are generally set by the specific product purpose and country-specific regulations. Alternative requirements will affect the optimal configuration and combination of operating parameters and ultimately the process performance. To illustrate these effects, Figures 9 and S10 and S11 show the Pareto fronts for the maximum productivity and minimum SEC under alternative constraints on CH_4_ purity and CH_4_ recovery for configuration 3 and for the other configurations, respectively (now again, *T*_0_ = 15 °C and ). These Pareto fronts are based on interpolated results (to limit the required number of simulations); therefore, we only discuss these qualitatively.

First, we consider increasingly strict constraints on CH_4_ purity (here, CH_4_ recovery ≥0.90), Figure 9a–c. On the one end, these reduce the maximum productivity (arrow I; left side of Figure 9a–b). This reduction is initially due to the smaller *Q*_in_ to move the CO_2_ adsorption front away from the column outlet and ultimately due to the longer *t*_FE_ to sharpen the CO_2_ adsorption front. Both reduce the amount of CO_2_ collected in the CH_4_ product but adversely affect the amount of CH_4_ product that is collected per cycle or per unit time (see also Figure S2). For maximum productivity, *P*_PR_ and *P*_BD_ are always near their upper and lower bounds, respectively. On the other end, stricter constraints on CH_4_ purity increase the minimum SEC (arrow II; bottom side of Figure 9a,c). This increase is due to (i) similarly the smaller *Q*_in_ and (ii) first the lower *P*_BD_ and then the higher *P*_PR_ that both increase the energy consumption. For minimum SEC, *t*_FE_ is mostly constant and approaches its upper bound. For intermediate productivity and SEC, all operating parameters vary interdependently.

In contrast, increasingly strict constraints on CH_4_ recovery (here, CH_4_ purity ≥0.90) have less of an effect on the trade-off between SEC and productivity, Figure 9d–f. As long as *P*_PR_ is not too high, high CH_4_ recovery is effectively provided “for free” by the pressure equalization steps. The maximum productivity is only reduced by the inability of higher *P*_PR_ to satisfy these increasingly strict constraints (arrow III). This inability is due to the lower selectivity of the sorbent at higher pressure.^51^ The reduction of the maximum productivity, however, merely shrinks the compatible domain but does not decrease the maximum productivity for a given SEC as such. Only the strictest constraint on CH_4_ recovery (here, 0.97) requires an increase of *Q*_in_ and *t*_FE_ and decrease of *P*_BD_ to provide a sharp CO_2_ adsorption front that displaces more CH_4_ from the column during the FE step.

### 3.5 Comparison with Alternative Adsorbents

The preceding analysis puts us in the position to compare the performance indicators for Cs-bentonite to those of alternative adsorbents. Before we do so, recall that all of the performance indicators are interdependent, i.e., within specific bounds, some performance indicators can be improved at the expense of others. This, together with varying biogas compositions, temperatures, and target product specifications between different studies, complicates a quantitative comparison to other published works.

Table 4 tabulates the performance indicators for Cs-bentonite for several different conditions and product purity and component recovery levels along the Pareto fronts in Figures 7a and 8c at the point where *P*_PR_ tends to 1 bar (except for the high productivity case). This point is often around the midpoint of the Pareto front. It is of further interest because a process at ambient pressure may as well reduce capital and maintenance costs due to a possible simplification of the unit, e.g., enabling the use of a blower or simply the overpressure from the digester instead of a compressor.

**Table 4:** Simulated Performance Indicators for Cs-Bentonite and for Several Conditions and Product Purity and Component Recovery Levels

CH_4_/CO_2_	*T*_0_ (°C)	C^a^	CH_4_ pur	CH_4_ rec	CO_2_ pur	CO_2_ rec	SEC^b^	prod^c^	notes
55/45	15	3	0.906	0.967	0.957	0.878	0.072	0.097	
55/45	25	3	0.904	0.964	0.952	0.874	0.071	0.082	higher temperature
65/35	15	3	0.904	0.964	0.926	0.809	0.046	0.115	larger CH_4_ feed fraction
55/45	15	7	0.908	0.981	0.975	0.878	0.079	0.055	high CH_4_ recovery
55/45	15	6	0.962	0.906	0.892	0.955	0.083	0.056	high CH_4_ purity
55/45	15	3	0.908	0.952	0.937	0.881	0.121	0.161	high productivity

a Configuration.

b Units: .

c Units: Nm_BG_^3^ kg^–1^ h^–1^.

In addition to Cs-bentonite, ref (51) also considered tetramethylammonium (TMA)-bentonite for biogas upgrading. However, in contrast to Cs-bentonite, the interlayer galleries of TMA-bentonite are accessible to CH_4_ and, therefore, this material shows a lower CO_2_/CH_4_ selectivity of ∼7 (Figure S12). In the Supporting Information, we provide a similar assessment for this material as for Cs-bentonite. We indeed find that its high CH_4_ adsorption capacity makes TMA-bentonite inappropriate for biogas upgrading (Figure S14).

By comparing our results in Table 4 with the results of the previous works in Table S1, we conclude the following. First, most of the previous works focused on high CH_4_ purity ≥0.97. While not the main focus of this work, by using CH_4_ product pressurization (i.e., configurations 5–6), also a CH_4_ purity of 0.962 was achieved (for a CH_4_ recovery of 0.906; higher CH_4_ purity is possible at the expense of CH_4_ recovery). On the other hand, the CH_4_ recovery obtained in this work is high compared to most of the previous works, with the notable exceptions of the dual-PSA units (refs (9 and 45)). We attribute this on the one hand to the relatively high CO_2_/CH_4_ selectivity up to ∼35 of Cs-bentonite and on the other hand to the use of the pressure equalization steps that were not used in some of the previous works. While not used in this work, product purge/rinse steps and/or dual-VPSA units to possibly further increase the CH_4_ purity and/or recovery should be the subject of a future study.

Second, the productivity of Cs-bentonite is mostly lower than or at most comparable to the conventional sorbents. This can mainly be attributed to its comparatively low CO_2_ adsorption capacity. However, two additional aspects should be noted here. (i) The duration of the PR, PPE, and RPE steps is in our simulations set to 1 min. The productivity can likely be increased by a reduction thereof, e.g., to ∼15–30 s for the PPE and RPE steps as in some previous works.^9,10,14,22,23,26,32,37,39,40,44,45^ (ii) To compare the required equipment size, the productivity can instead be expressed per unit sorbent volume. Sorbents with a high volumetric adsorption capacity (i.e., high adsorption capacity and particle density) are then favored. On the one hand, the particle density of Cs-bentonite (∼1400 kg m^–3^) is comparable to some zeolites,^10,22,31,34^ silica gel,^47^ and MOF-508b.^10^ On the other hand, it is (significantly) higher than, e.g., that of some other zeolites,^9,13,24,25,28,33^ MOFs,^46,49^ porous polymeric beads,^44^ silicalites,^8^ and CMS.^10−12,14,25,36−43^

Third, the SEC is always significantly lower for Cs-bentonite than for the conventional sorbents. We attribute this to the CO_2_ adsorption isotherms that are relatively linear in the relevant pressure domain. They thereby facilitate the sorbent regeneration already under weak vacuum conditions. Indeed, the benefits of sorbents with relatively linear CO_2_ adsorption isotherms for their easy regeneration (or the reverse) were suggested in various previous studies.^7,10,16,22,25,36,44,45,47,49,50^

Thus, Cs-bentonite generally shows excellent performance as compared to the conventional sorbents. These alternative materials often suffer from a trade-off between high CO_2_/CH_4_ selectivity and easy regeneration.^12,16,49,70^ (For example, AC shows easy regeneration but low CO_2_/CH_4_ selectivity ≲5,^12,16−19^ whereas zeolite 13X shows higher CO_2_/CH_4_ selectivity, but its regeneration is impeded by its rather steep CO_2_ adsorption isotherms.^10,16,25,26,28,29,36^) Therefore, we attribute the excellent performance of Cs-bentonite to the rather unique combination of high CO_2_/CH_4_ selectivity and easy regeneration.

## 4 Conclusions

The simulations presented in this work demonstrate the ability of Cs-bentonite to separate the CH_4_ and CO_2_ in biogas (i.e., biogas upgrading). A sufficiently high CH_4_ purity for grid injection in, e.g., The Netherlands, and a high CH_4_ recovery can be reached at significantly lower specific energy consumption than for conventional adsorbent materials. This is even possible at ambient feed pressure, without product purge and rinse steps, and by using a single upgrading stage. The high CH_4_ recovery and low specific energy consumption are due to (i) the high CO_2_/CH_4_ selectivity and (ii) the linear CO_2_ adsorption isotherms that facilitate the regeneration under weak vacuum conditions at ambient temperature. In the case of high CH_4_ recovery, a CO_2_ product with a typical purity of ∼0.93–0.97 is coproduced. Such a CO_2_ product can, for example, be used for CO_2_ sequestration to actually produce carbon-negative bio-CH_4_.^44^

The process performance depends crucially on the VPSA cycle configuration and the operating parameters that should, therefore, be tailored toward the specific requirements on product purity and component recovery. Pressure equalization steps between multiple columns are essential for high CH_4_ recovery but should be avoided when high CH_4_ recovery is not required. For CH_4_ recovery ≥0.95, a two-column system with one PE step is desired. Bio-CH_4_ product refluxes do not significantly improve the productivity and specific energy consumption for the production of bio-CH_4_ with a purity ≳0.90. However, bio-CH_4_ product refluxes can improve these performance indicators when higher CH_4_ purity is required and/or increase the maximum attainable CH_4_ purity. Also, the operating conditions affect the process performance; smaller CO_2_ fractions in the feed biogas (i.e., within the typical range of biogas composition) and lower temperatures (i.e., within the typical range of ambient temperatures) increase the productivity and/or decrease the specific energy consumption.

Interestingly, for most operating conditions and constraints on product purity and component recovery, there exists a domain along the (productivity-specific energy consumption) Pareto front in which the process does not require feed pressures above atmospheric. This enables the use of a rather simple unit to reduce capital and maintenance cost (e.g., not requiring a compressor). However, the current work did not take into account the pressurization of the CH_4_ product that is required for certain downstream applications. Should this be accounted for, then elevated feed pressures may be desirable to increase productivity as the CH_4_ product is then available at that pressure.

The current work provides a strong case for using Cs-bentonite in biogas upgrading and discusses several aspects that should be taken into account when designing a VPSA unit to do so. The design and commissioning of a pilot plant that is based on the current simulation in- and outputs should test this material on a larger scale with actual biogas and provide input parameters for further process development. We hope that, ultimately, the use of low-cost and low specific energy consumption sorbent materials like bentonite results in the wider use of biogas upgrading for the energy and chemical transitions and beyond.

## Supporting Information Available

Previously reported performance indicators for various sorbent materials; physical properties of gas components and mixtures and heat and mass transport coefficients; details of the model implementation; Langmuir adsorption isotherms fit parameters; schematic of process cycle configurations; effect of individual operating parameters; parity plots simulation and interpolation; effect of RPE direction; multidimensional sensitivity analyses; Pareto fronts under alternative recovery and purity constraints; assessment of tetramethylammonium-bentonite (PDF)
