# Supplementary material for: Cs-Bentonite Clay for Biogas Upgrading: A Numerical Assessment
Source: Ind Eng Chem Res. 2025 Apr 10;64(16):8359–74. doi: 10.1021/acs.iecr.4c04491 (PMC12022990; doi:10.1021/acs.iecr.4c04491)
Supplement: Supplementary file 1 — ie4c04491_si_001.pdf [file ie4c04491_si_001.pdf]

Supporting Information

Cs-bentonite Clay for Biogas Upgrading: A Numerical Assessment

Niels Mendel,[*,†] *Jordanus J. P. (Jordi) Boon,*[‡] *Igor Sîrețanu,*[†] *Frieder Mugele*[†]*, and Derk W. F. (Wim) Brilman*[‡]

[†]Physics of Complex Fluids, Faculty of Science and Technology, MESA+ Institute for Nanotechnology, University of Twente, P.O. Box 217, Enschede 7500 AE, The Netherlands

[‡]Sustainable Process Technology, Faculty of Science and Technology, University of Twente, P.O. Box 217, Enschede 7500 AE, The Netherlands

Corresponding author e-mail: n.mendel@utwente.nl

Contents:

- Previously reported performance indicators for various sorbent materials (**Table S1)**
- Physical properties of gas components (**Table S2**) and mixtures, and heat and mass transport coefficients
- Details of the model implementation
- Langmuir adsorption isotherms fit parameters (**Table S3-S4**)
- Schematic of process cycle configurations (**Figure S1**)
- Effect of individual operating parameters (**Figure S2**, **Table S5**)
- Parity plots simulation and interpolation (**Figure S3**)
- Effect of RPE direction (**Figure S4**)
- Multi-dimensional sensitivity analyses (**Figure S5-S9**)
- Pareto fronts under alternative recovery and purity constraints, configurations 1-2, 4-7 (**Figure S10-S11**)
- Assessment of tetramethylammonium-bentonite (**Figure S12-S14**)

Table S1. Previously reported performance indicators for various sorbent materials.[a]

Ref.	Adsorbent	CH_4/CO_2	CH_4 Purity	CH_4 Recovery	SEC[b]	Prod.[c]
1	Zeolite 5A	60/40	0.988	0.994	0.24	0.51
2	Silica gel	55/45	0.980	0.973	-	-
3	Zeolite 13X	67/33	0.98	0.85	0.422	-
4	Zeolite 13X	60/40	0.99	0.93	0.223	0.396
5	Zeolite 13X	67/33	0.992	0.850	-	0.243
6	CMS-3K + Zeolite 13X	55/45	0.980	0.803	-	0.246
3	CMS-3K	67/33	0.98	0.85	0.214	-
7	CMS-3K	60/40	0.97	0.80	0.48	0.065
		60/40	0.97	0.95	0.28	0.039
8	CMS-3K	55/45	0.981	0.799	-	0.131
9	CMS KP 407	60/40	0.972	0.935	-	0.108
		60/40	0.994	0.838	-	0.161
3	MOF-508b	67/33	0.98	0.85	0.185	-
10	MIL-53(Al)	40/60	0.994	0.928	0.720	0.133
11	Porours Polymeric Beads	60/40	0.924	0.908	0.17	-
12	Silica gel	60/40	0.98	0.823	-	0.346
13	Silicalite	62/38	0.978	0.585	-	0.703
		50/50	0.981	0.520	-	0.736

[a] Specific energy consumption and productivity are recalculated to match the units and definitions used in this work. [b] Units: kWh $Nm^{-3}_{CH_4}$; [c] Units: Nm^3_{BG} kg^{-1} h^{-1}.

Physical properties of gas components and mixtures and heat and mass transport coefficients

The physical properties of the different gas species are listed in **Table S2**. Herein, and in the equations below, m_i is the molecular mass, M_i is the molar mass, σ_i is the collision diameter, ϵ_i is the Lennard-Jones interaction strength parameter, $c_{\mathrm{P},i}$ is the heat capacity at constant pressure, and y_i is the gas fraction, all of component i. k_B is Boltzmann's constant and γ is the adiabatic constant.

Table S2. Physical properties of the different gas species.[14]

Property	CO_2	CH_4	N_2	Units
M	$44.01 \cdot 10^{-3}$	$16.04 \cdot 10^{-3}$	$28.01 \cdot 10^{-3}$	$\mathrm{kg\ mol^{-1}}$
σ	$3.996 \cdot 10^{-10}$	$3.780 \cdot 10^{-10}$	$3.667 \cdot 10^{-10}$	m
ϵ/k_B	190	154	99.8	K
c_P	37.2	35.6	39.1	$\mathrm{J\ mol^{-1}\ K^{-1}}$
γ	1.3	1.3	1.3	-

The density of the gas mixture ρ_g is calculated from its composition, **Eq. S1**.

$$\rho_\mathrm{g} = \frac{P}{RT}\sum_i y_i M_i \tag{S1}$$

The gas component contribution to the viscosity $\mu_{\mathrm{g},i}$ is calculated using Chapman-Enskog theory, **Eq. S2**.[14]

$$\mu_{\mathrm{g},i} = \frac{5}{16}\left(\frac{m_i kT}{\pi}\right)^{\frac{1}{2}} \frac{1}{\sigma_i^2 \Omega_{\mu,i}} \tag{S2}$$

Herein, Ω_μ is the dimensionless collision integral for viscosity, curve fitted by Neufeld *et al*, **Eqs. S3-S4**.[14,15]

$$\Omega_{\mu,i} = \frac{1.16145}{\left(T_{ii}^*\right)^{0.14874}} + \frac{0.52487}{\exp\left(0.77320 \cdot T_{ii}^*\right)} + \frac{2.16178}{\exp\left(2.43787 \cdot T_{ii}^*\right)} \tag{S3}$$

$$T_{ij}^* = T \cdot \left[\left(\frac{\epsilon}{k_\mathrm{B}}\right)_i \left(\frac{\epsilon}{k_\mathrm{B}}\right)_j\right]^{-\frac{1}{2}} \tag{S4}$$

The gas component contribution to the thermal conductivity $\lambda_{g,i}$ is calculated from the Eucken approximation, **Eq. S5**.[14]

$$\lambda_{\mathrm{g},i} = \left(\frac{c_{\mathrm{P},i}}{M_i} + \frac{5}{4}\frac{k_\mathrm{B}}{m_i}\right)\mu_{\mathrm{g},i} \tag{S5}$$

The mixing rules proposed by Wilke[14,16] are used to calculate the viscosity and the thermal conductivity of the gas mixture, **Eq. S6**, with $f \in \{\mu, \lambda\}$.

$$f_\mathrm{g} = \sum_i \frac{y_i f_{\mathrm{g},i}}{\sum_j y_j \Phi_{ij}}, \text{ with } \Phi_{ij} = \frac{1}{\sqrt{8}}\left(1 + \frac{m_i}{m_j}\right)^{-\frac{1}{2}}\left[1 + \left(\frac{\mu_i}{\mu_j}\right)^{\frac{1}{2}}\left(\frac{m_j}{m_i}\right)^{\frac{1}{4}}\right]^2 \tag{S6}$$

The mass diffusivity for binary mixtures ($i \neq j$) and self-diffusivity ($i = j$) are approximated using Chapman-Enskog theory, **Eq. S7**.[14]

$$D_{ij}^\mathrm{m} = \frac{3}{8}\left[\frac{1}{2\pi}\left(\frac{1}{m_i} + \frac{1}{m_j}\right)\right]^{\frac{1}{2}}\left[\frac{(\sigma_i + \sigma_j)}{2}\right]^{-2}\frac{(k_\mathrm{B}T)^{\frac{3}{2}}}{\Omega_{D,ij}P} \tag{S7}$$

Here, Ω_D is the dimensionless collision integral for diffusion, curve fitted by Neufeld *et al*, **Eq. S8**.[14,15]

$$\Omega_{D,ij} = \frac{1.06036}{\left(T_{ij}^*\right)^{0.15610}} + \frac{0.19300}{\exp\left(0.47635 \cdot T_{ij}^*\right)} + \frac{1.03587}{\exp\left(1.52996 \cdot T_{ij}^*\right)} + \frac{1.76474}{\exp\left(3.89411 \cdot T_{ij}^*\right)} \tag{S8}$$

The axial mass dispersion coefficient is estimated by **Eq. S9**.[17]

$$\epsilon_\mathrm{b} D_\mathrm{ax}^\mathrm{M} = D_{ij}^\mathrm{m}[20 + 0.5 \cdot \mathrm{Sc{\cdot}Re}] \tag{S9}$$

Herein, the binary diffusivity for CO_2 and CH_4 is used (i.e., D_{ij}^m with i CO_2 and j CH_4). The effective mass diffusivity of gas species i within the particle, $D_{\mathrm{m},i}^\mathrm{eff}$ is estimated from a combination of molecular self-diffusivity (**Eq. S7**, for which the average total pressure within the particle pores is used) and Knudsen diffusivity (**Eq. S11**)[14] following the Bosanquet equation, **Eq. S10**.[18]

$$D_{\mathrm{m},i}^\mathrm{eff} = \frac{\epsilon_\mathrm{p}}{\tau}D_{\mathrm{m},i} = \frac{\epsilon_\mathrm{p}}{\tau}\left(\frac{1}{D_{ii}^\mathrm{m}} + \frac{1}{D_i^\mathrm{K}}\right)^{-1} \tag{S10}$$

$$D_i^\mathrm{K} = \frac{d_\mathrm{m}}{3}\sqrt{\frac{8k_\mathrm{B}T}{\pi m_i}} \tag{S11}$$

Here, ϵ_P is the particle porosity, τ is the particle tortuosity and d_m is the typical pore diameter. MMT and bentonite (particles) are known to feature wide and hierarchical (multi-modal) pore size and pore-throat size distributions.[19] Therefore, we estimate an effective pore diameter d_m (and the adsorption LDF coefficient $k_{c,i}$ in **Eq. 6**) from experimental breakthrough- and desorption measurements, **Figure 4**. In lieu of a better estimate, we set $\tau = 2.0$. The error introduced herein is essentially included in the effective pore diameter.

The film mass transfer coefficient (**Eq. S12**; therein, D_{ij}^{m} with i CO_2 and j CH_4),[17] the axial heat dispersion coefficient (**Eq. S13**),[20] and the wall heat transfer coefficient (**Eq. S14**)[21] are estimated based on correlations proposed in literature.

$$k_f = D_{ij}^{m}/d_p\left[2 + 1.1 \cdot \mathrm{Sc}^{1/3} \cdot \mathrm{Re}^{0.6}\right] \quad \text{(S12)}$$

$$D_{ax}^{T} = \lambda_g[7 + 0.5 \cdot \mathrm{Pr} \cdot \mathrm{Re}] \quad \text{(S13)}$$

$$h_w = \lambda_g/d_r\,[12.5 + 0.048 \cdot \mathrm{Re}] \quad \text{(S14)}$$

Herein, d_p and d_r are the particle and column diameter, respectively. The dimensionless variables are defined as $\mathrm{Re} = \rho_g|v_s|d_p/\mu_g$, $\mathrm{Sc} = \mu_g/\left(\rho_g D_{ax}^{M}\right)$, $\mathrm{Pr} = \sum_i\left(y_i c_{P,i}/M_i\right)\mu_g/\lambda_g$, and $\mathrm{Bi}_i = k_f r_p/D_{m,i}^{eff}$. Herein, $|v_s|$ is the local superficial velocity and r_p is the particle radius. Gas properties at the cell edges that are required to calculate transfer coefficients at the cell edges (e.g., D_{ax}^{M}, D_{ax}^{T}, **Eq. 8**; see below for the discretization scheme) are assumed to be the average of those in the surrounding nodes or equal to those in the nearest node in the case of the boundaries.

Details of the model implementation

To solve the system of PDEs, the column was divided into $N = 30$ cells with indices $n = [1..30]$, each with a length Δz. Cell-averaged values are denoted f_n. Fluxes are calculated at the cell edges. The quantities at the (downstream) cell edge, denoted $f_{n+0.5}$ for steps executed in the forward direction, is related to the cell-averaged value f_n using a three-point Weighted Essentially Non-Oscillatory (WENO) scheme (**Eqs. S15-S17**).

$$f_{n+0.5} = \frac{\alpha_n}{\alpha_n + \beta_n}\left[\frac{1}{2}(f_n + f_{n+1})\right] + \frac{\beta_n}{\alpha_n + \beta_n}\left[\frac{3}{2}(f_n - f_{n-1})\right] \tag{S15}$$

$$\alpha_n = \frac{2}{3}(f_{n+1} - f_n + \delta)^{-4} \tag{S16}$$

$$\beta_n = \frac{1}{3}(f_n - f_{n-1} + \delta)^{-4} \tag{S17}$$

Here, $\delta = 10^{-10}$. An analogous scheme is used for steps executed in the 'backward' direction. This scheme is further detailed in Reference 22. A first order spatial derivative in cell n is converted to an algebraic expression by using a finite volume scheme, **Eq. S18**.

$$\left[g\frac{\partial f}{\partial z}\right]_n = g_n\left(\frac{f_{n+0.5} - f_{n-0.5}}{\Delta z}\right) \tag{S18}$$

Here, f and g can be (a product of) quantities and/or properties. (First order spatial derivatives at the cell edges, for example to calculate the local superficial velocity v_s at the cell edge from the local pressure gradient at the cell edge, are approximated similarly with $n \rightarrow n + 0.5$.) Any second order spatial derivative in cell n is approximated by using a combination of a finite difference and a finite volume scheme, **Eq. S19**.

$$\left[\frac{\partial}{\partial z}\left(g\frac{\partial f}{\partial z}\right)\right]_n = \frac{1}{\Delta z}\left[g_{n+0.5}\left(\frac{f_{n+1} - f_n}{\Delta z}\right) - g_{n-0.5}\left(\frac{f_n - f_{n-1}}{\Delta z}\right)\right] \tag{S19}$$

A half-cell approximation $f_1 - f_0 = 2(f_1 - f_{0.5})$ and similarly for $f_{N+0.5}$ is used near the column boundaries.[22] The values at the column boundaries, denoted as $f_{0.5} = f|_{z=0}$ and $f_{N+0.5} = f|_{z=L_r}$, are set by the boundary conditions.

Table S3. Single-site Langmuir model fit parameters of the isotherms in **Figure 3** and **Figure S12**.

**Sorbent**	**Gas**	$n_{i,1}$ mol kg^{-1}	$b_{0,i,1}$ bar^{-1}	$\Delta H_{i,1}$ kJ mol^{-1}	R^2
Cs-b.	CO_2	1.188	$3.18 \cdot 10^{-6}$	-31.18	0.9891
	CH_4	0.379	$1.96 \cdot 10^{-3}$	-9.029	0.9814
TMA-b.	CO_2	1.057	$5.09 \cdot 10^{-6}$	-30.48	0.9865
	CH_4	0.939	$9.85 \cdot 10^{-5}$	-19.22	0.9963

Table S4. Dual-site Langmuir model fit parameters of the isotherms in **Figure 3** and **Figure S12**.

**Sorbent**	**Gas**	$n_{i,1}$ mol kg^{-1}	$b_{0,i,1}$ bar^{-1}	$\Delta H_{i,1}$ kJ mol^{-1}	$n_{i,2}$ mol kg^{-1}	$b_{0,i,2}$ bar^{-1}	$\Delta H_{i,2}$ kJ mol^{-1}	R^2
Cs-b.	CO_2	0.801	$1.29 \cdot 10^{-6}$	-35.32	1.11	$6.92 \cdot 10^{-6}$	-21.83	0.9993
TMA-b.	CO_2	0.559	$5.25 \cdot 10^{-6}$	-33.37	0.832	$3.01 \cdot 10^{-6}$	-26.74	0.9994
	CH_4	0.184[a]	$1.75 \cdot 10^{-5}$[a]	-27.34[a]	0.937	$1.17 \cdot 10^{-4}$	-17.01	0.9977

[a] Unusually large confidence interval.

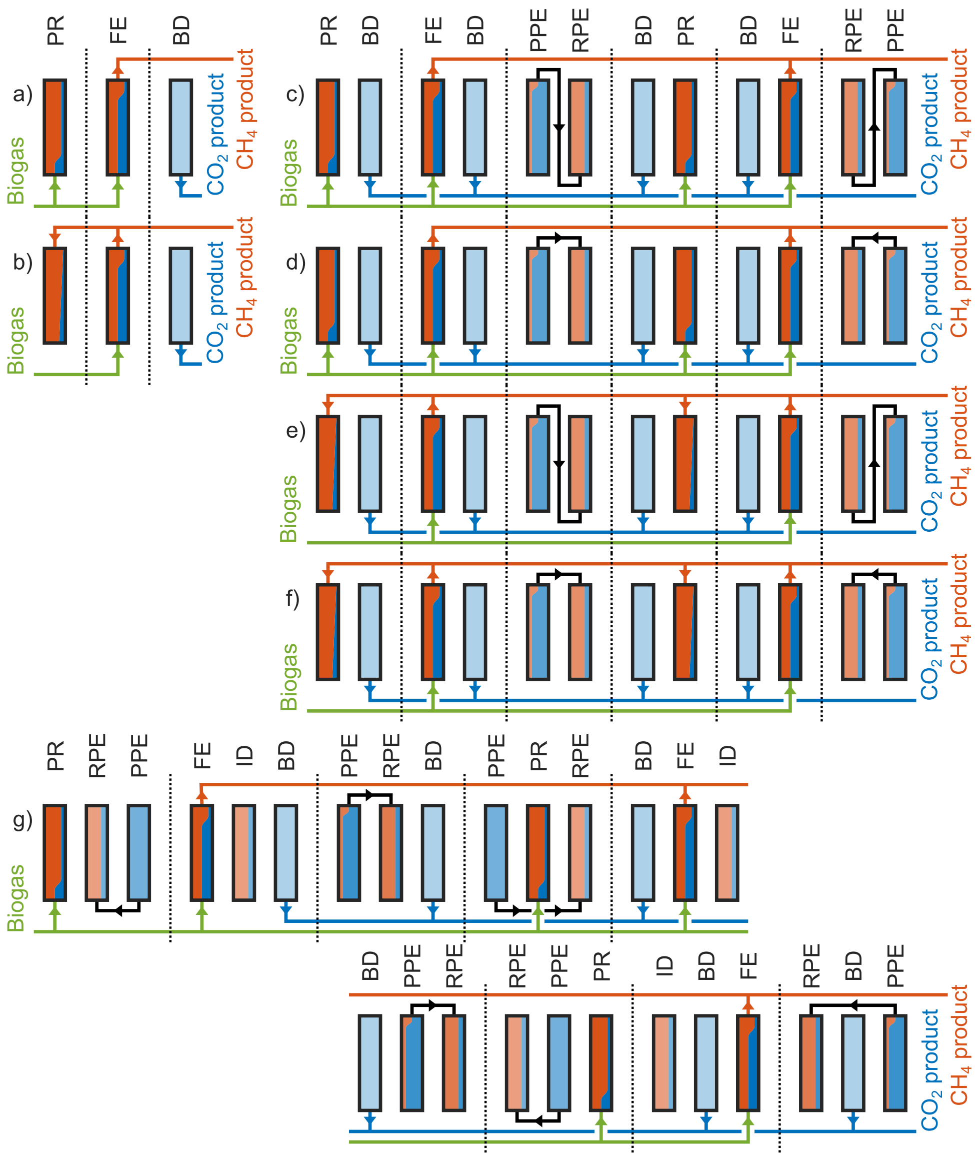

Figure S1. Schematic illustration of the process cycle configurations, with (a-g) configuration 1-7. The colors inside the column illustrate the typical component concentration in the column voids at the end of the respective step. Red: CH_4; blue: CO_2; deeper colors indicate higher pressures.

Effect of individual operating parameters

The effect of the individual operating parameters on (i) the adsorbed CO_2 at the end of each step along the axial dimension (as in **Figure 5**e) and (ii) the output gas flows over time (as in **Figure 5**c) is illustrated in **Figure S2**. The corresponding performance indicators are listed in **Table S5**. These effects, while all other operating parameters are fixed, can be summarized as follows. First, higher P_{PR} result in higher CO_2 adsorption at the end of each step and larger output flow rates during the FE (CH_4 only), PPE and BD steps. Consequently, higher P_{PR} result in increased productivity, however at the expense of increased SEC for compression. Higher P_{PR} also increase the amount of adsorbed CH_4 and the amount of CH_4 and CO_2 in the column voids at the end of the FE step. Moreover, the CO_2 adsorption front at the end of the FE step also shifts slightly toward the column outlet with increasing P_{PR} due to the larger amount CO_2 that is already provided in the PR and RPE steps. This shift suggests that the optimum Q_{in} can shift accordingly with P_{PR}. These effects also imply that the effect of P_{PR} on the CH_4 purity and the CH_4 recovery is non-trivial. In **Table S5**, we find that higher P_{PR} result in increased CH_4 purity at the expense of CH_4 recovery. Second, higher P_{BD} decrease the output CO_2 flow during the BD step and increase the CO_2 retention at the end of the BD step and the output CO_2 flow during the FE step. Due to the then lower working capacity of the column, the CH_4 output flow during the FE step is also reduced. In turn, the amount of CH_4 collected in the CO_2 product is nearly independent of P_{BD}. Consequently, higher P_{BD} result in decreased CH_4 purity, CH_4 recovery, and productivity. The SEC, however, then also decreases because less work is needed to evacuate the column. Third, longer t_{FE} decrease all output flow rates during the FE and BD steps and, as a result of the then reduced flow velocity, sharpen the CO_2 adsorption front. In addition, longer t_{FE} decrease the CO_2 retention at the end of the BD step (see P_{BD}) due to the incomplete regeneration of the sorbent at very short t_{BD}. Consequently, longer t_{FE} increase CH_4 purity at the expense of productivity. Fourth, higher Q_{in} shift the CO_2 adsorption front at the

end of the FE step toward or beyond the outlet of the column (i.e., CO_2 eventually shows breakthrough). The CH_4 output flow rate during the FE step is also increased due to the higher utilization of the column while t_{FE} is fixed. In turn, the CH_4 collected in the CO_2 product during the BD step decreases with increasing Q_{in}. This is due to more CH_4 being displaced from the column during the FE step. Consequently, higher Q_{in} result in increased CH_4 recovery at the expense of CH_4 purity, and in increased productivity and decreased SEC. Together, the effects of the individual operating parameters demonstrate that, ultimately, the process performance depends on the specific combination of operating parameters that is used. Some of these effects also suggest a trade-off between performance indicators (e.g., CH_4 purity versus CH_4 recovery for Q_{in}, SEC versus productivity for P_{PR} and P_{BD}). Moreover, the (optimal) operating parameters are likely interdependent (e.g., the suggested shift of Q_{in} with P_{PR}). Thus, one-dimensional sensitivity analyses do likely not provide the optimal combination of operating parameters.

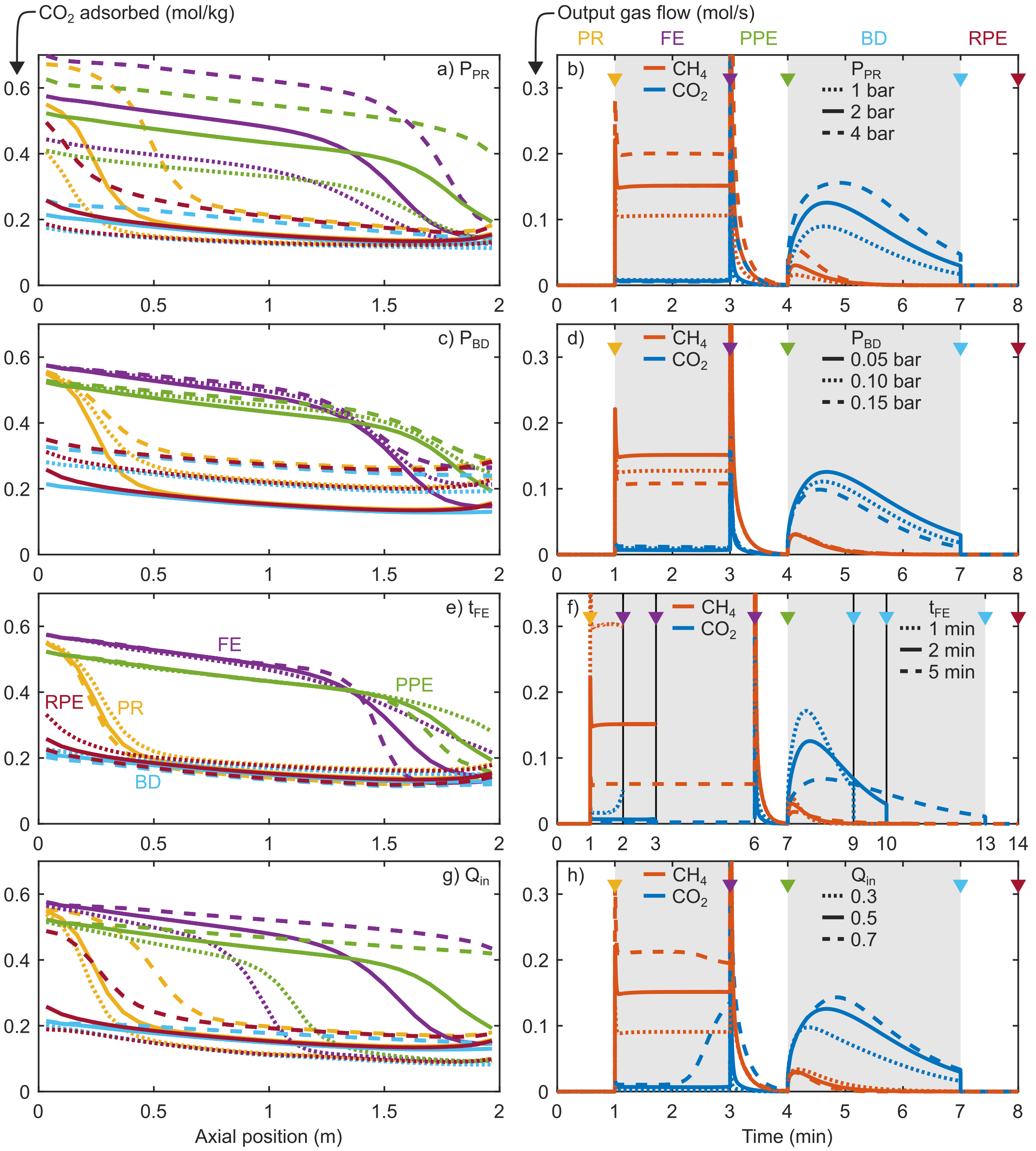

Figure S2. Simulation results (CSS) of configuration 3, $y_{CO_2}^{BG} = 0.45$, $T_0 = 15°C$, and variable operating parameter operating parameters (a-b) P_{PR}, (c-d) P_{BD}, (e-f) t_{FE}, and (g-h) Q_{in} while all other operating parameters are fixed at $P_{PR} = 2$ bar, $P_{BD} = 0.05$ bar, $t_{FE} = 2$ min, and $Q_{in} = 0.5$. Panel (a,c,e,g) CO_2 adsorbed along the axial dimension at the end of each step. Panel (b,d,f,h) output gas flows over time. Steps are indicated by the alternating background colors in (b,d,f,h). Line colors in (a,c,e,g) correspond to the color of the downward triangles in (b,d,f,h). The solid lines are as in **Figure 5**.

Table S5. Performance indicators for the simulations in **Figure S2**.

P_{PR} (bar)	P_{BD} (bar)	t_{FE} (min)	Q_{in} (-)	CH_4 pur.	CH_4 rec.	SEC[a]	Productivity[b]
1	0.05	2	0.5	0.929	0.950	0.076	0.078
2	0.05	2	0.5	0.957	0.939	0.111	0.113
4	0.05	2	0.5	0.971	0.921	0.158	0.153
2	0.05	2	0.5	0.957	0.939	0.111	0.113
2	0.10	2	0.5	0.930	0.923	0.097	0.097
2	0.15	2	0.5	0.901	0.905	0.088	0.084
2	0.05	1	0.5	0.935	0.940	0.113	0.151
2	0.05	2	0.5	0.957	0.939	0.111	0.113
2	0.05	5	0.5	0.963	0.936	0.108	0.065
2	0.05	2	0.3	0.974	0.856	0.120	0.074
2	0.05	2	0.5	0.957	0.939	0.111	0.113
2	0.05	2	0.7	0.851	0.965	0.100	0.152

[a] Units: kWh $Nm^{-3}_{CH_4}$; [b] Units: Nm^3_{BG} kg^{-1} h^{-1}.

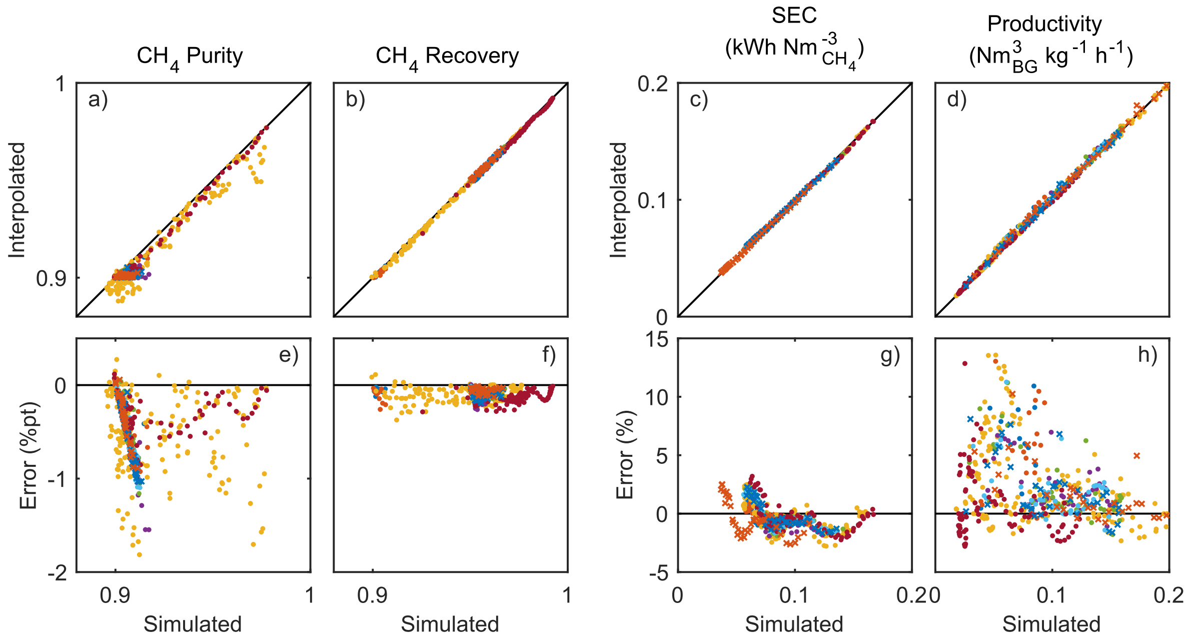

Figure S3. Parity plots between the interpolated performance indicators (ordinate in (a-d); based on the initial seed simulations only) and simulated performance indicators (abscissa). (e-h) indicate the deviation of the interpolated performance indicators from the simulated performance indicators.

Effect of RPE direction

The effect of the direction of the RPE step is illustrated in **Figure S4**. The direction of the RPE step has limited effects on the SEC and productivity, but does affect the CH_4 purity and (to a lesser extent) the CH_4 recovery. A backward RPE step (i.e., counter-current to the biogas FE step; configurations 4 and 6) is preferred over a forward RPE step (configuration 3 and 5) when P_{BD} is high, P_{PR} is low, and/or Q_{in} is small. The inflow gas during the RPE step then contains relatively little CO_2 and/or the partial pressure of CO_2 at the end of the BD step is then relatively large. If this is the case, then the provided gas can desorb and/or displace part of the CO_2 near the column end where the CH_4 product is collected. This increases the CH_4 purity and is similar to a PR step using the CH_4 product (configurations 2, 5 – 6), or a CH_4 product purge used in previous works.[1-3,5,8-10,13,23-25] If instead P_{BD} is low, P_{PR} is high, and/or Q_{in} is large, then a backward RPE step increases the CO_2 partial pressure at the end of the column where the CH_4 product is collected and thereby reduces CH_4 purity (e.g., Santos *et al.*[5]).

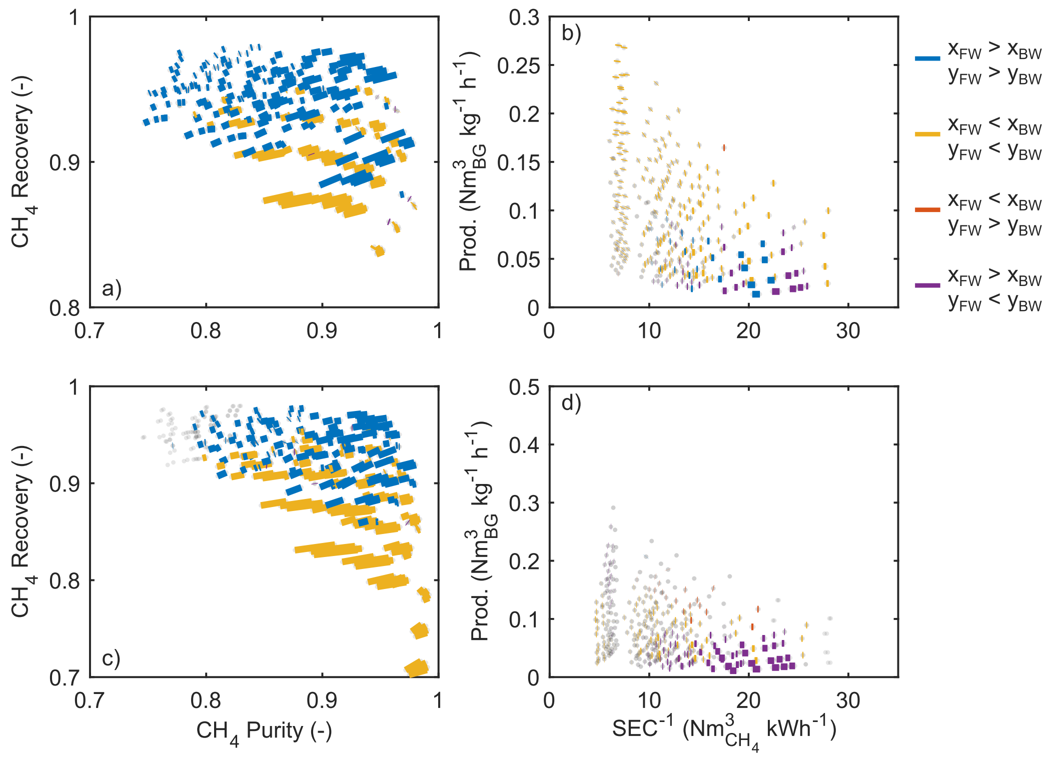

Figure S4. The effect of the direction of the receive pressure equalization step (FW: forward, co-current with respect to the biogas feed; BW: backward, counter-current with respect to the biogas feed) on the performance indicators, for (a-b) configurations 3 (FW) and 4 (BW), and (c-d) configurations 5 (FW) and 6 (BW). Points connected by the colored lines have the same operating parameters. The line color indicates the performance difference (see legend), where x is CH_4 purity or SEC^{-1} and y is CH_4 recovery or productivity.

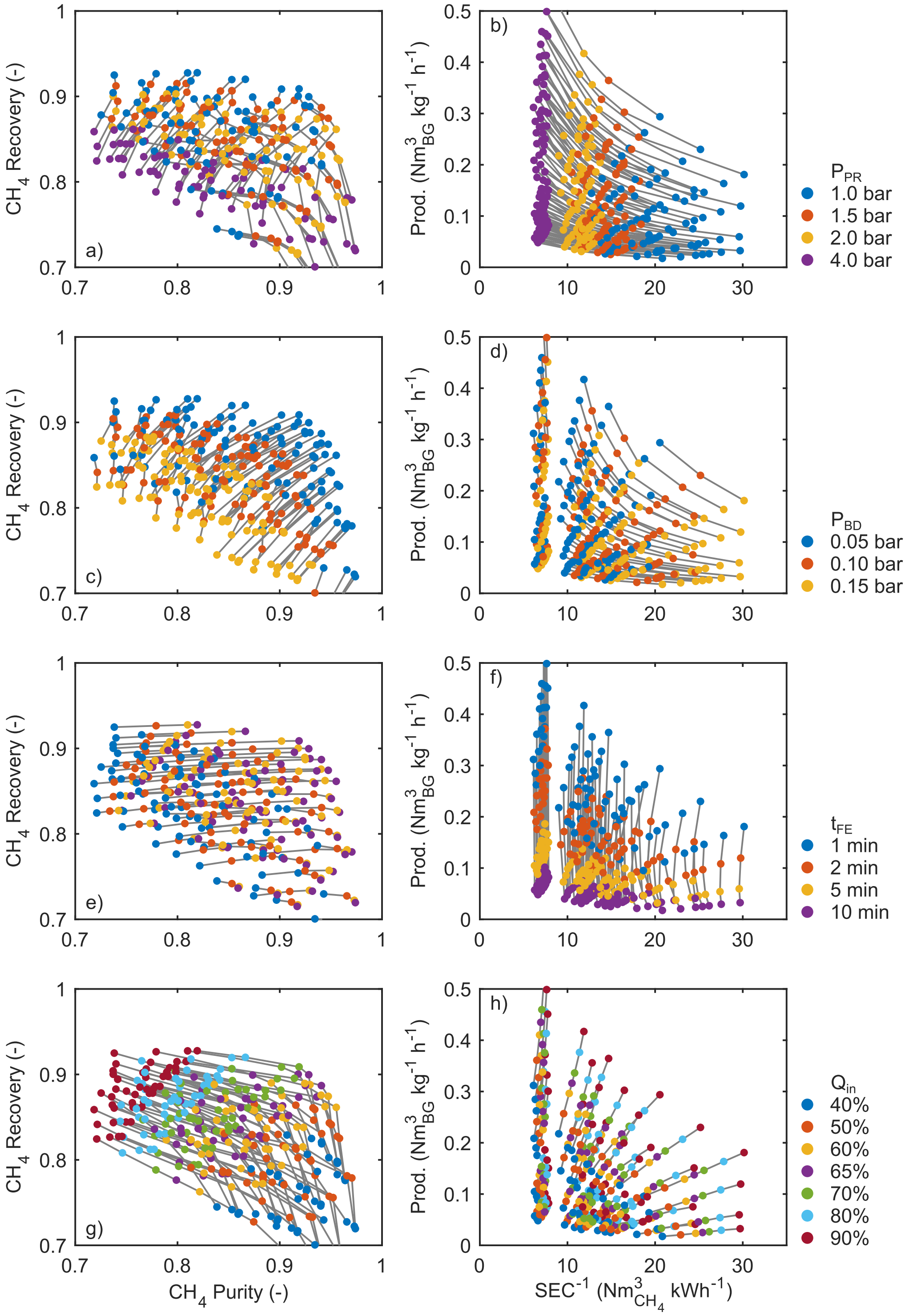

Figure S5. Multi-dimensional sensitivity analyses (configuration 1; $y_{CO_2}^{BG} = 0.45$, $T_0 = 15$°C). Different colors indicate variable (a-b) P_{PR}, (c-d) P_{BD}, (e-f) t_{FE}, and (g-h) Q_{in}. Points connected by a solid grey line have all other operating parameters fixed.

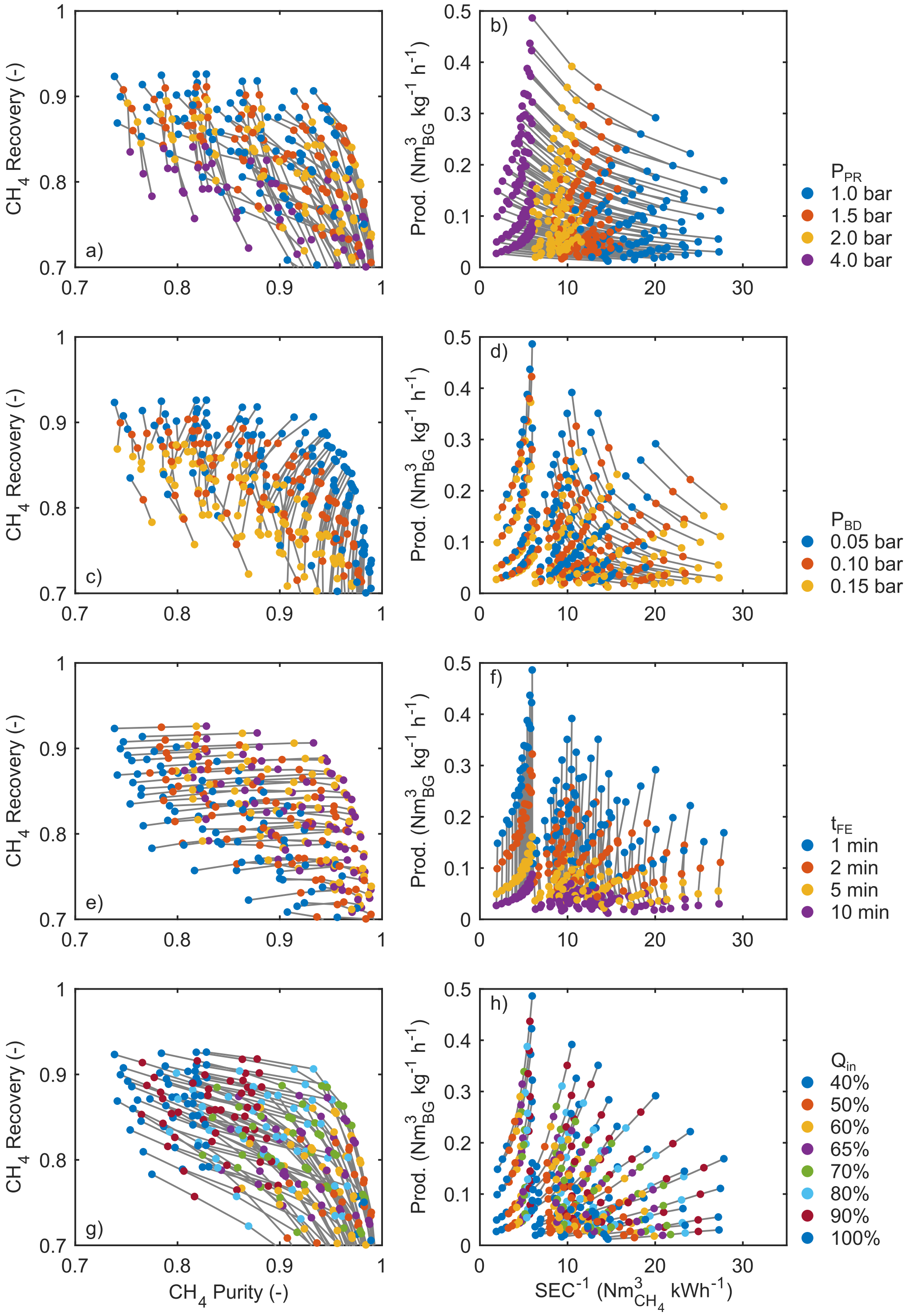

Figure S6. Multi-dimensional sensitivity analyses (configuration 2; $y_{CO_2}^{BG} = 0.45$, $T_0 = 15$°C). Different colors indicate variable (a-b) P_{PR}, (c-d) P_{BD}, (e-f) t_{FE}, and (g-h) Q_{in}. Points connected by a solid grey line have all other operating parameters fixed.

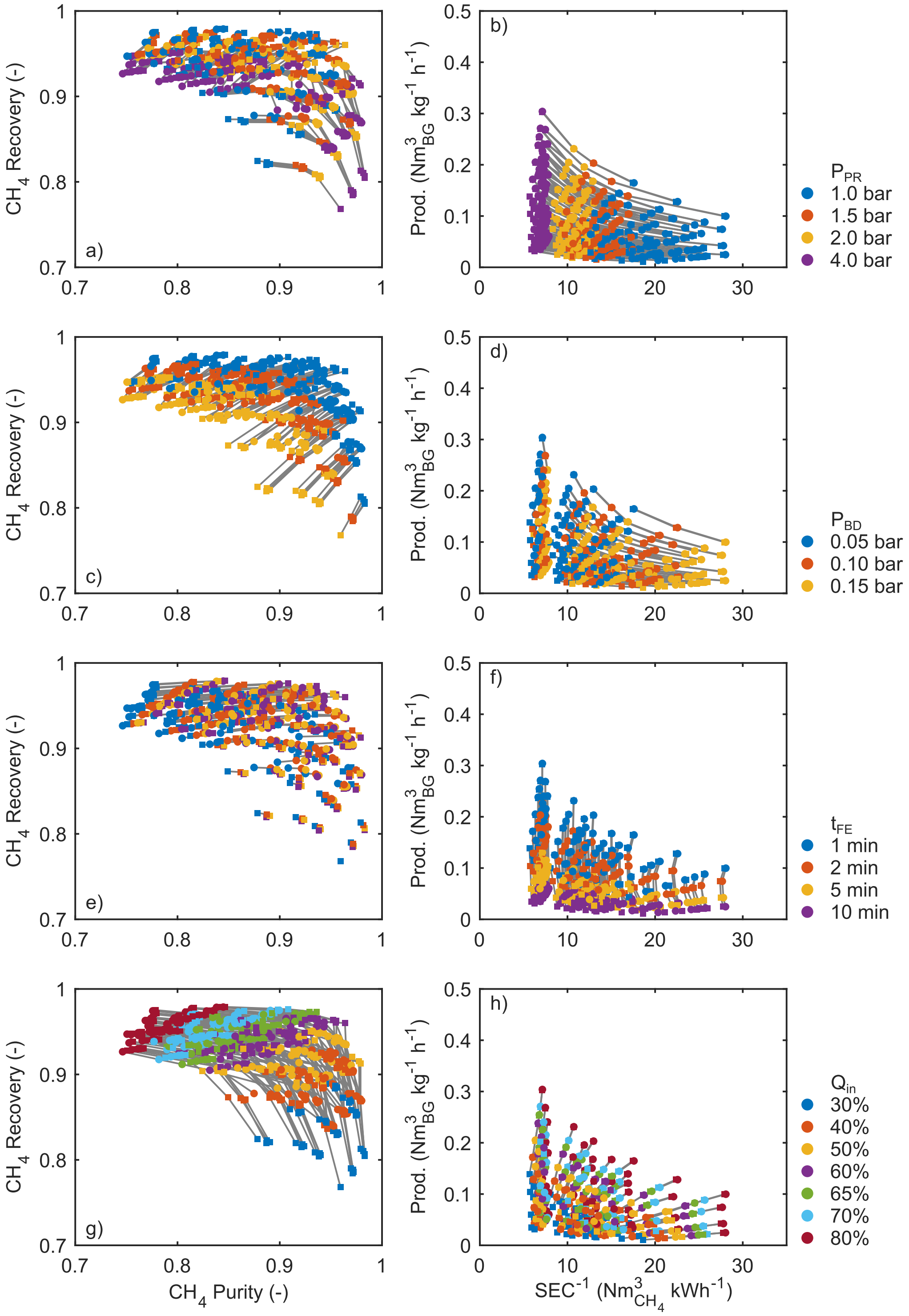

Figure S7. Multi-dimensional sensitivity analyses (configurations 3 [squares] and 4 [circles]; $y_{CO_2}^{BG} = 0.45$, $T_0 = 15°C$). Different colors indicate variable (a-b) P_{PR}, (c-d) P_{BD}, (e-f) t_{FE}, and (g-h) Q_{in}. Points connected by a solid grey line have all other operating parameters fixed.

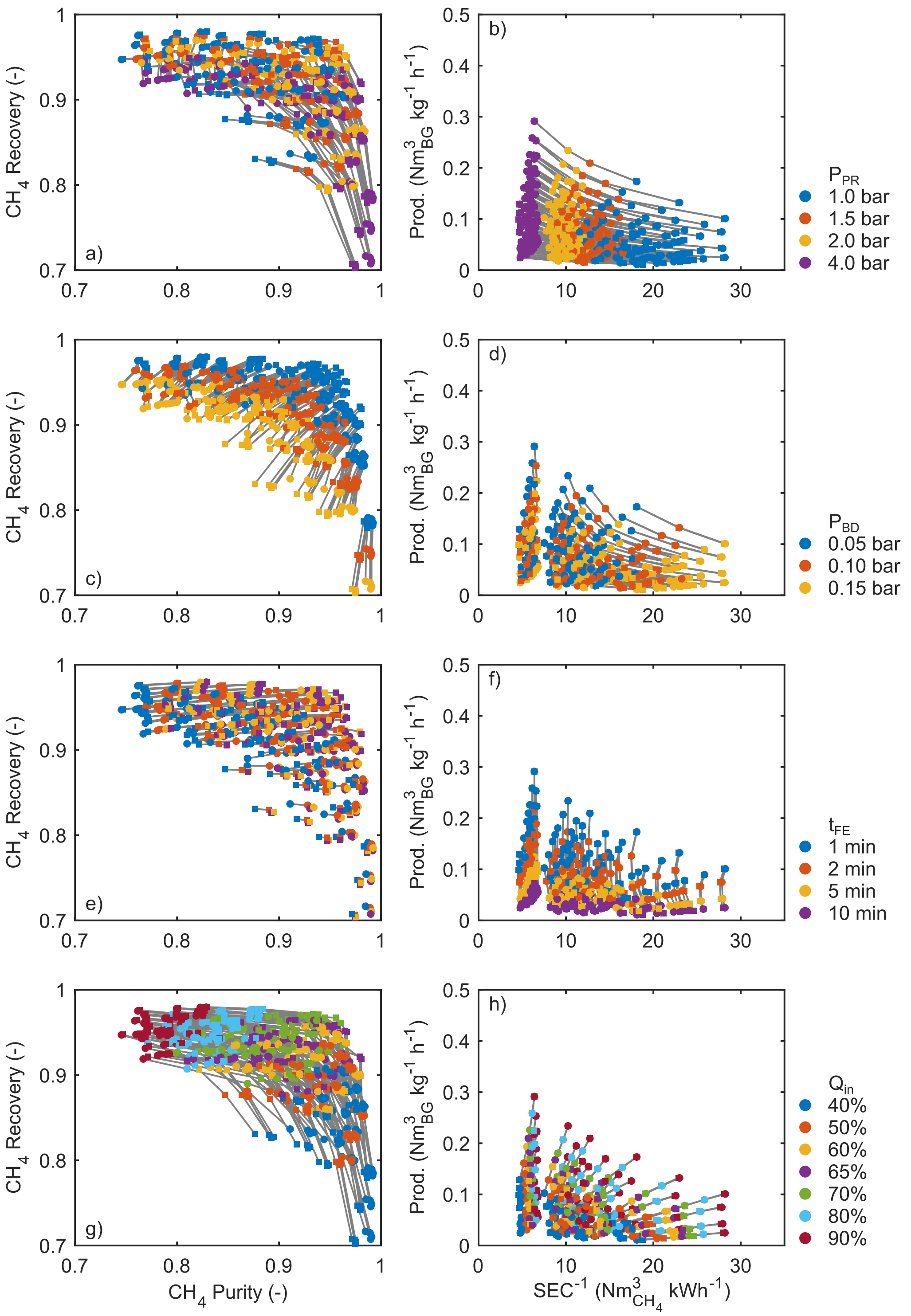

Figure S8. Multi-dimensional sensitivity analyses (configurations 5 [squares] and 6 [circles]; $y_{CO_2}^{BG} = 0.45$, $T_0 = 15°C$). Different colors indicate variable (a-b) P_{PR}, (c-d) P_{BD}, (e-f) t_{FE}, and (g-h) Q_{in}. Points connected by a solid grey line have all other operating parameters fixed.

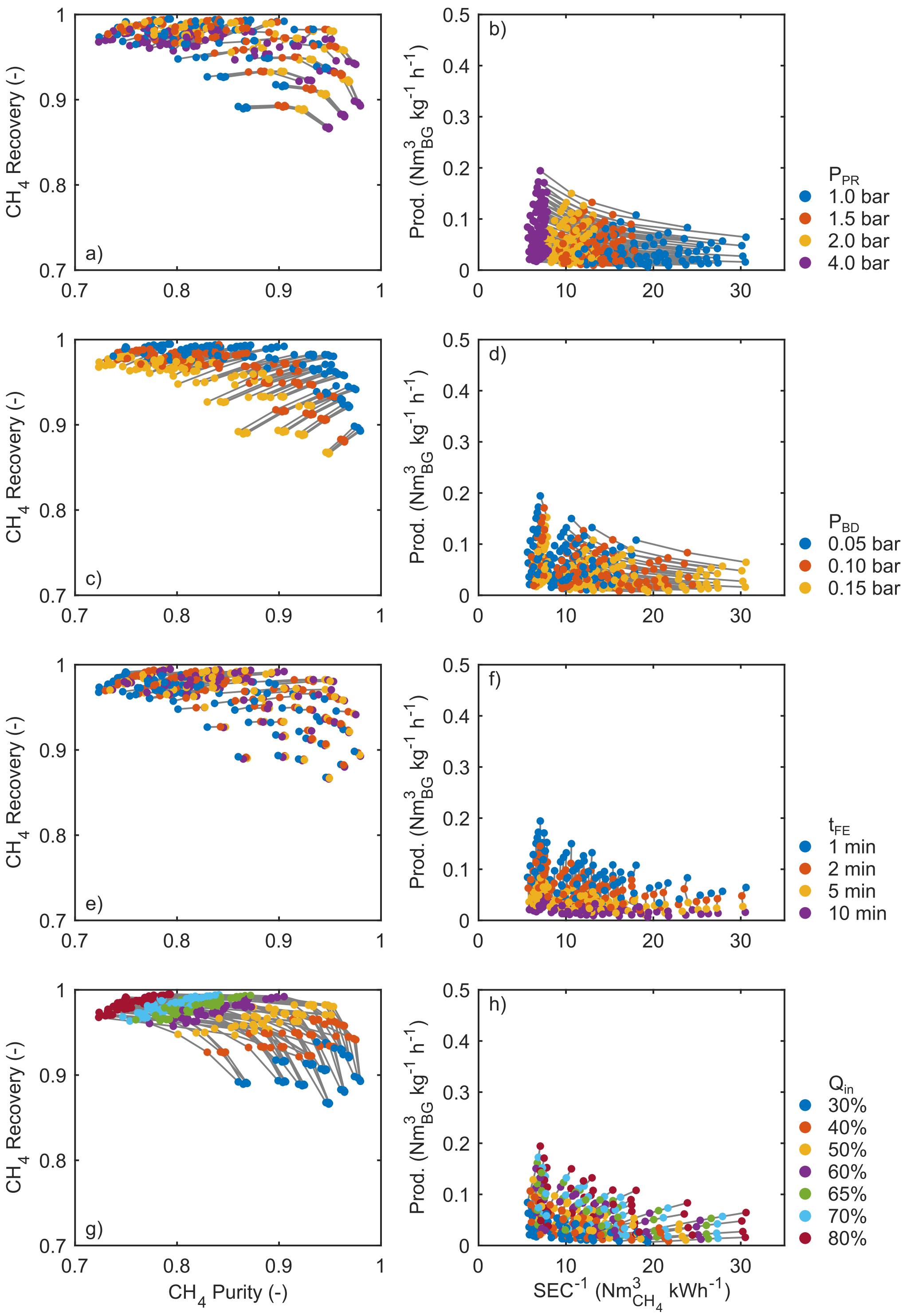

Figure S9. Multi-dimensional sensitivity analyses (configuration 7; $y_{CO_2}^{BG} = 0.45$, $T_0 = 15°C$). Different colors indicate variable (a-b) P_{PR}, (c-d) P_{BD}, (e-f) t_{FE}, and (g-h) Q_{in}. Points connected by a solid grey line have all other operating parameters fixed.

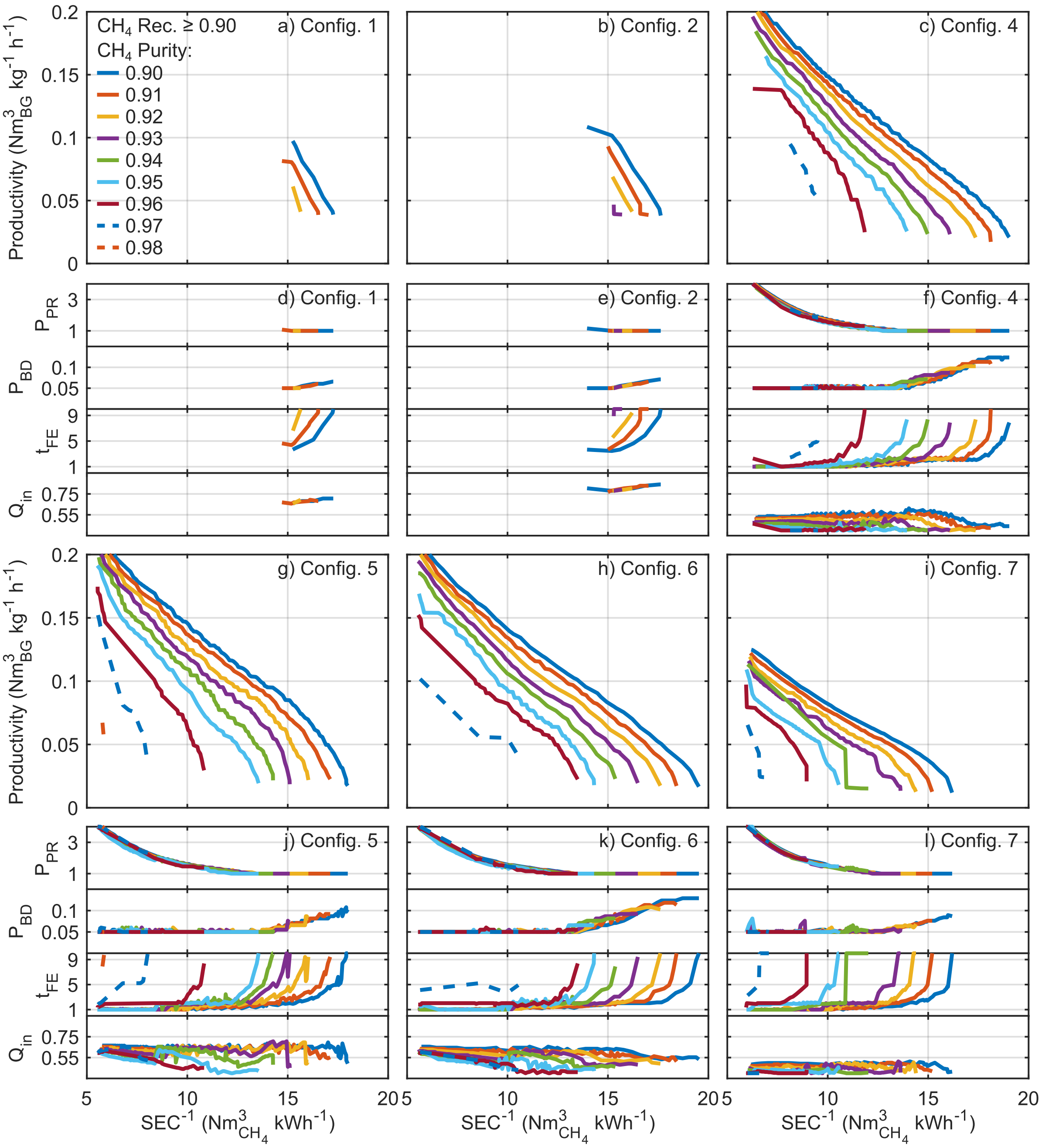

Figure S10. Interpolated Pareto fronts for the maximum productivity and minimal SEC under the constraints CH_4 recovery ≥ 0.90 and variable minimal CH_4 purity for (a) configuration 1, (b) configuration 2, (c) configuration 4, (g) configuration 5, (h) configuration 6, and (i) configuration 7. (d-f,j-l) Operating parameters along the Pareto fronts in (a-c,g-i), respectively. $T_0 = 15°C$; $y_{CO_2}^{BG} = 0.45$.

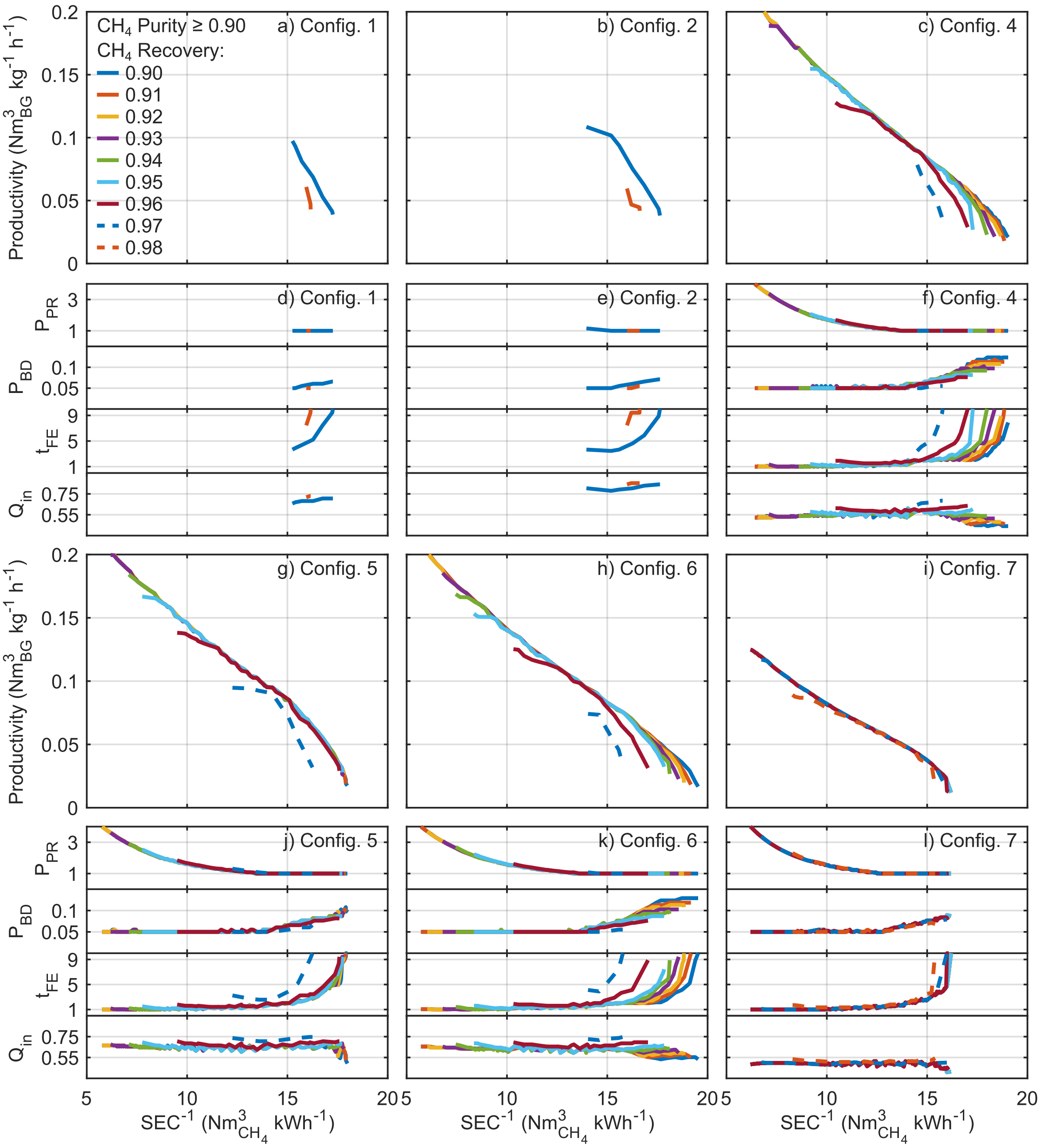

Figure S11. Interpolated Pareto fronts for the maximum productivity and minimal SEC under the constraints CH_4 purity ≥ 0.90 and variable minimal CH_4 recovery for (a) configuration 1, (b) configuration 2, (c) configuration 4, (g) configuration 5, (h) configuration 6, and (i) configuration 7. (d-f,j-l) Operating parameters along the Pareto fronts in (a-c,g-i), respectively. $T_0 = 15°C$; $y_{CO_2}^{BG} = 0.45$.

Tetramethylammonium-bentonite

Other than Cs-bentonite, Ref. 26 suggested using tetramethylammonium (TMA^+)-exchanged bentonite for biogas upgrading, albeit most likely with poorer performance than Cs-bentonite. We assess the performance of TMA-bentonite in this Section. This requires two adaptations to the model. First, the adsorption isotherms. The experimental adsorption isotherms of CO_2 and CH_4 on TMA-bentonite are presented and discussed Ref. 26. We fit both sets of isotherms with the temperature-dependent single-site Langmuir (SSL) and dual-site Langmuir (DSL) models as are discussed in **Section 2.3**, as presented in **Figure S12**. The fit parameters are presented in **Table S3** and **Table S4**.

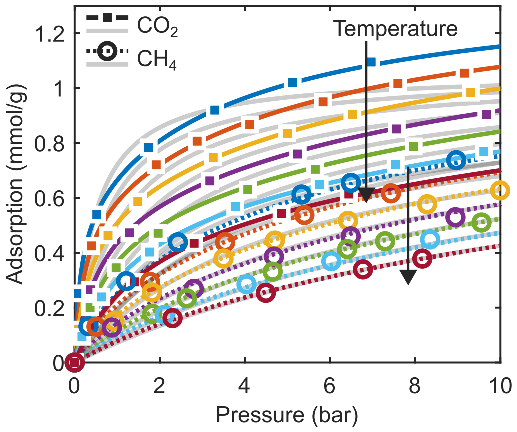

Figure S12. Adsorption isotherms of CO_2 and CH_4 on TMA-bentonite. Arrows indicate increasing temperature from 10°C to 70°C at 10°C increments. The experimental data is presented with solid squares (CO_2) and open circles (CH_4). The temperature-dependent fits are presented with colored solid lines (CO_2; DSL) colored dashed lines (CH_4; DSL) and grey lines (SSL).

We use the $\Delta H_{i,1}$ fitted using the SSL model as an effective enthalpy of adsorption for the energy balance and the DSL model to describe the equilibrium adsorption. For CH_4, this is different than for Cs-bentonite, because the interlayer galleries of TMA-bentonite are accessible to CH_4.[26-30]

Second, competitive adsorption. In contrast to Cs-bentonite, competitive effects between the adsorption of CO_2 and CH_4 are in fact significant for TMA-bentonite.[26] Under the assumption that CH_4 can only be outcompeted by CO_2 (and not the reverse), we adapt the model for this bentonite by substituting in **Eq. 6** $q^*_{\mathrm{CH_4}} \rightarrow q^{*\prime}_{\mathrm{CH_4}}$, where we define $q^{*\prime}_{\mathrm{CH_4}}$ as in **Eq. S20**.

$$q^{*'}_{\mathrm{CH_4}} = q^*_{\mathrm{CH_4}} \cdot \left[1 - \frac{q_{\mathrm{CO_2}}}{1.057\ \mathrm{mol\ kg^{-1}}}\right] \tag{S20}$$

Here, $1.057\ \mathrm{mol\ kg^{-1}}$ is the CO_2 adsorption capacity of TMA-bentonite fitted using the SSL model. In other words, we scale the adsorption capacity for CH_4 with (an estimate for) the fraction of unoccupied CO_2 adsorption sites.

Now, to validate the model adapted to TMA-bentonite, we again compare the output flow compositions of experimental (cyclic) breakthrough and regeneration (in N_2) measurements[26] with simulated output flow compositions under the same conditions, **Figure S13**. The properties of the TMA-bentonite particles are set equal to those of the Cs-bentonite particles, **Table 2**. The simulated outputs describe the experimental outputs well, provided that the competitive adsorption is included in the model (see the difference between the black and yellow lines). Specifically, more CH_4 is desorbed and displaced upon the progression of the CO_2 adsorption front than simulated using the model without competitive adsorption.

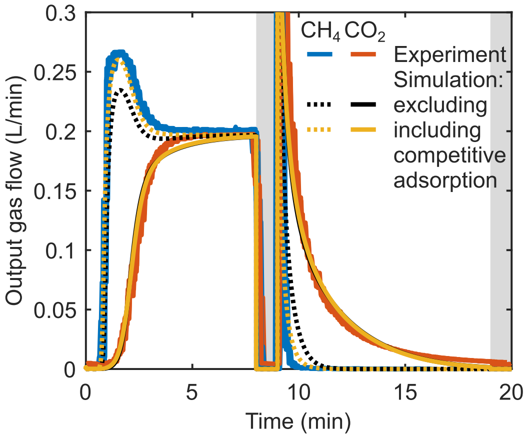

Figure S13. Comparison between experimental[26] and simulated cyclic breakthrough- and regeneration measurements on 50 g of TMA-bentonite particles. Column dimensions: $d_{\mathrm{r}} =$

1.3 cm and $L_r = 60$ cm. Each cycle consisted of (i) a 8 minute forward feed step with an inlet gas mixture containing only CO_2 (mole fraction of 0.50 ± 0.02) and CH_4 (mole fraction of 0.50 ± 0.02) at a total flow rate of $0.40\ \mathrm{L\ min^{-1}}$, (ii) a 1 minute forward idle step (grey background), (iii) a 10 minute forward feed step with N_2 (for regeneration) at a flow rate of $0.60\ \mathrm{L\ min^{-1}}$, and (iv) a 1 minute forward idle step (grey background). The volumetric flow rates are actual values. The experimental cycles are shifted on top of each other.

To assess TMA-bentonite for biogas upgrading, we performed additional simulations using configuration 3 under the conditions $T_0 = 15°\mathrm{C}$ and $y_{\mathrm{CO_2}}^{\mathrm{BG}} = 0.45$ and using a column of the same dimensions and mass loading as in the main text. **Figure S14** displays for all simulated combinations of operating parameters and for both TMA- and Cs-bentonite the component recovery as a function of the component purity (similar to **Figure 6**). The maximum CH_4 purity and CO_2 recovery are nearly independent of the sorbent material used, however, for TMA-bentonite only at much lower CH_4 recovery. For a minimal CH_4 purity of 0.9, the maximum CH_4 recovery is significantly lower for TMA-bentonite (~0.874) than for Cs-bentonite (~0.977). The much lower CH_4 recovery for TMA-bentonite is due to the higher CH_4 adsorption thereon, compare **Figure S12** and **Figure 3**.[26] Thus, its high CH_4 adsorption capacity makes TMA-bentonite inappropriate for this separation.

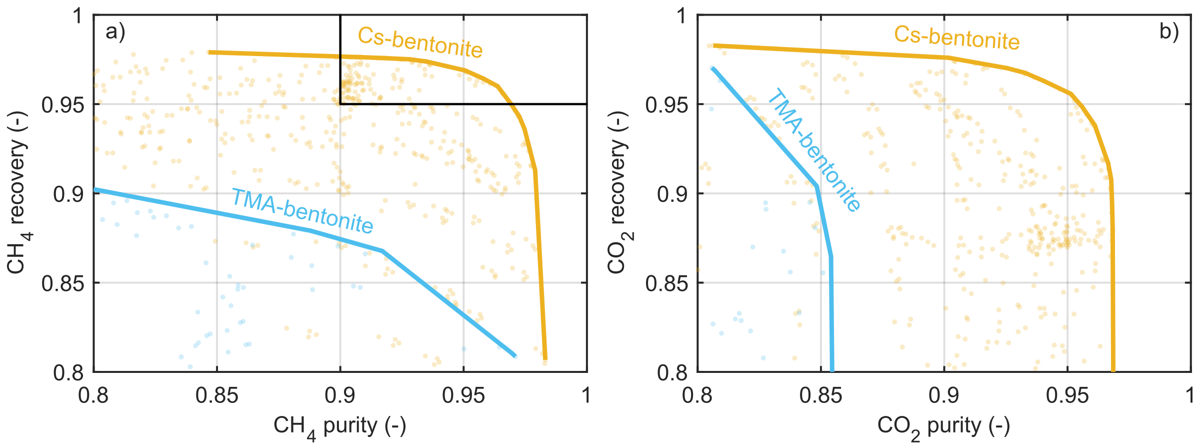

Figure S14. Simulated (opaque dots) and maximum (solid lines) component recovery as a function of component purity for (a) CH_4 and (b) CO_2. Configuration 3; $T_0 = 15°C$; $y_{CO_2}^{BG} = 0.45$.

References

1. Augelletti, R.; Conti, M.; Annesini, M.C., *Pressure swing adsorption for biogas upgrading. A new process configuration for the separation of biomethane and carbon dioxide.* J. Cleaner Prod., **2017**, *140*, 1390-1398.
2. Shen, Y.; Shi, W.; Zhang, D.; Na, P.; Fu, B., *The removal and capture of CO_2 from biogas by vacuum pressure swing process using silica gel.* J. CO2 Util., **2018**, *27*, 259-271.
3. Wu, B.; Zhang, X.; Xu, Y.; Bao, D.; Zhang, S., *Assessment of the energy consumption of the biogas upgrading process with pressure swing adsorption using novel adsorbents.* J. Cleaner Prod., **2015**, *101*, 251-261.
4. Khunpolgrang, J.; Yosantea, S.; Kongnoo, A.; Phalakornkule, C., *Alternative PSA process cycle with combined vacuum regeneration and nitrogen purging for CH_4/CO_2 separation.* Fuel, **2015**, *140*, 171-177.
5. Santos, M.S.; Grande, C.A.; Rodrigues, A.r.E., *Pressure swing adsorption for biogas upgrading. Effect of recycling streams in pressure swing adsorption design.* Ind. Eng. Chem. Res., **2011**, *50*, 974-985.
6. Grande, C.A.; Rodrigues, A.E., *Layered vacuum pressure-swing adsorption for biogas upgrading.* Ind. Eng. Chem. Res., **2007**, *46*, 7844-7848.
7. Chouikhi, N.; Brandani, F.; Pullumbi, P.; Perre, P.; Puel, F., *Biomethane production by adsorption technology: New cycle development, adsorbent selection and process optimization.* Adsorption, **2020**, *26*, 1275-1289.
8. Grande, C.A.; Rodrigues, A.E., *Biogas to fuel by vacuum pressure swing adsorption I. Behavior of equilibrium and kinetic-based adsorbents.* Ind. Eng. Chem. Res., **2007**, *46*, 4595-4605.
9. Canevesi, R.L.S.; Andreassen, K.A.; Silva, E.A.; Borba, C.E.; Grande, C.A., *Evaluation of simplified pressure swing adsorption cycles for bio-methane production.* Adsorption, **2019**, *25*, 783-793.
10. Ferreira, A.F.P.; Ribeiro, A.M.; Kulaç, S.; Rodrigues, A.E., *Methane purification by adsorptive processes on MIL-53 (Al).* Chem. Eng. Sci., **2015**, *124*, 79-95.
11. Golmakani, A.; Nabavi, S.A.; Manović, V., *Production of negative-emission biomethane by twin double-bed pressure swing adsorption with tail gas sequestration.* Chem. Eng. J., **2021**, *408*, 127312.
12. Grande, C.A.; Morence, D.G.B.; Bouzga, A.M.; Andreassen, K.A., *Silica gel as a selective adsorbent for biogas drying and upgrading.* Ind. Eng. Chem. Res., **2020**, *59*, 10142-10149.
13. Delgado, J.A.; Uguina, M.A.; Sotelo, J.L.; Ruiz, B.; Gomez, J.M., *Fixed-bed adsorption of carbon dioxide/methane mixtures on silicalite pellets.* Adsorption, **2006**, *12*, 5-18.
14. Bird, R.B., *Transport phenomena.* Appl. Mech. Rev., **2002**, *55*, R1-R4.
15. Neufeld, P.D.; Janzen, A.R.; Aziz, R.A., *Empirical equations to calculate 16 of the transport collision integrals Ω(l, s)* for the Lennard-Jones (12–6) potential.* J. Chem. Phys., **1972**, *57*, 1100-1102.
16. Wilke, C.R., *A viscosity equation for gas mixtures.* J. Chem. Phys., **1950**, *18*, 517-519.
17. Wakao, N.; Funazkri, T., *Effect of fluid dispersion coefficients on particle-to-fluid mass transfer coefficients in packed beds: correlation of Sherwood numbers.* Chem. Eng. Sci., **1978**, *33*, 1375-1384.
18. Shafeeyan, M.S.; Daud, W.M.A.W.; Shamiri, A., *A review of mathematical modeling of fixed-bed columns for carbon dioxide adsorption.* Chem. Eng. Res. Des., **2014**, *92*, 961-988.

19. Kuila, U.; Prasad, M., *Specific surface area and pore-size distribution in clays and shales.* Geophys. Prospect., **2013**, *61*, 341-362.
20. Grande, C.A.; Rodrigues, A.E., *Propane/propylene separation by pressure swing adsorption using zeolite 4A.* Ind. Eng. Chem. Res., **2005**, *44*, 8815-8829.
21. Dantas, T.L.P.; Amorim, S.M.; Luna, F.M.T.; Silva Jr, I.J.; de Azevedo, D.C.S.; Rodrigues, A.E.; Moreira, R.F.P.M., *Adsorption of carbon dioxide onto activated carbon and nitrogen-enriched activated carbon: surface changes, equilibrium, and modeling of fixed-bed adsorption.* Sep. Sci. Technol., **2009**, *45*, 73-84.
22. Haghpanah, R.; Majumder, A.; Nilam, R.; Rajendran, A.; Farooq, S.; Karimi, I.A.; Amanullah, M., *Multiobjective optimization of a four-step adsorption process for postcombustion CO_2 capture via finite volume simulation.* Ind. Eng. Chem. Res., **2013**, *52*, 4249-4265.
23. Canevesi, R.; Grande, C.A., *Biogas upgrading by pressure swing adsorption using zeolite 4A. Effect of purge on process performance.* Sep. Purif. Technol., **2023**, *309*, 123015.
24. Kim, Y.J.; Nam, Y.S.; Kang, Y.T., *Study on a numerical model and PSA (pressure swing adsorption) process experiment for CH_4/CO_2 separation from biogas.* Energy, **2015**, *91*, 732-741.
25. Cavenati, S.; Grande, C.A.; Rodrigues, A.E., *Upgrade of methane from landfill gas by pressure swing adsorption.* Energy Fuels, **2005**, *19*, 2545-2555.
26. Mendel, N.; Sîretanu, I.; Mugele, F.; Brilman, D.W.F., *Biogas Upgrading Using Cation-Exchanged Bentonite Clay.* Ind. Eng. Chem. Res., **2023**, *62*, 17883-17892.
27. Barrer, R.M.; MacLeod, D.M., *Activation of montmorillonite by ion exchange and sorption complexes of tetra-alkyl ammonium montmorillonites.* Trans. Faraday Soc., **1955**, *51*, 1290-1300.
28. Bowers, G.M.; Loring, J.S.; Walter, E.D.; Burton, S.D.; Bowden, M.E.; Hoyt, D.W.; Arey, B.; Larsen IV, R.K.; Kirkpatrick, R.J., *Influence of smectite structure and hydration on supercritical methane binding and dynamics in smectite pores.* J. Phys. Chem. C, **2019**, *123*, 29231-29244.
29. Ziemiański, P.P.; Derkowski, A.; Szczurowski, J.; Kozieł, M., *The structural versus textural control on the methane sorption capacity of clay minerals.* Int. J. Coal Geol., **2020**, *224*, 103483.
30. Ziemiański, P.P.; Derkowski, A.; Szczerba, M.; Guggenheim, S., *Smectite crystallite swelling under high pressure of methane.* J. Phys. Chem. C, **2021**, *125*, 7598-7610.
